# Untargeted Metabolomics of Dairy Cows as Influenced by the Combinations of Essential Oil Blends and Fumaric Acid as Natural Feed Additives Using RUSITEC

**DOI:** 10.3390/metabo15100681

**Published:** 2025-10-21

**Authors:** Joel O. Alabi, Deborah O. Okedoyin, Michael Wuaku, Chika C. Anotaenwere, Oludotun O. Adelusi, Kelechi A. Ike, DeAndrea Gray, Olatunde A. Oderinwale, James M. Enikuomehin, Kingsley A. Ekwemalor, Yewande O. Fasina, Hamid D. Ismail, Ahmed E. Kholif, Uchenna Y. Anele

**Affiliations:** 1 Department of Animal Sciences, North Carolina Agricultural and Technical State University, Greensboro, NC 27411, USA; joalabi@aggies.ncat.edu (J.O.A.); dookedoyin@aggies.ncat.edu (D.O.O.); mwuaku@aggies.ncat.edu (M.W.); ccanotaenwere@aggies.ncat.edu (C.C.A.); ooadelusi@aggies.ncat.edu (O.O.A.); kaike@aggies.ncat.edu (K.A.I.); dgray3@aggies.ncat.edu (D.G.); oaoderinwale@aggies.ncat.edu (O.A.O.); jmenikuomehin@aggies.ncat.edu (J.M.E.); kaekwemalor@ncat.edu (K.A.E.); yfasina@ncat.edu (Y.O.F.); aekholif@ncat.edu (A.E.K.); 2 Department of Computational Data Science and Engineering, North Carolina Agricultural and Technical State University, Greensboro, NC 27411, USA; hdismail@ncat.edu

**Keywords:** dairy cows, essential oils, fumarate, RUSITEC, metabolome, metabolic pathways

## Abstract

Background and Objectives: The potential of essential oils (EOs) and fumaric acid (FA) to modulate ruminal fermentation and mitigate greenhouse gas emissions in dairy cows has attracted significant attention. However, little is known about the specific metabolites produced as a result of their interaction. This study investigated the combined effects of essential oil blends (EOBs) and FA on rumen metabolites using a rumen simulation technique (RUSITEC) system. Materials and Methods: Three rumen-cannulated, non-lactating Holstein Friesian cows served as inoculum donors. The total mixed ration (TMR; CON) comprised corn silage (60%), alfalfa hay (20%), and concentrate (20%). Three distinct EOBs were formulated: EOB1 [Garlic, Lemongrass, Cumin, Lavender, and Nutmeg; at 4:2:2:1:1, respectively], EOB2 [Anise, Clove, Oregano, Cedarwood, and Ginger; at 4:2:2:1:1, respectively], and EOB3 [Clove, Anise, Peppermint, and Oregano; at 4:3:2:1, respectively]. Four treatments evaluated were control (CON), EFA1 (EOB1 + FA), EFA2 (EOB2 + FA), and EFA3 (EOB3 + FA). EOBs and FA were included at 10 µL/g feed and 3% of TMR, respectively. Rumen effluents were collected over 5 days for metabolome analysis using liquid chromatography-mass spectrometry (LC–MS). Results: A total of 661 metabolites were detected and identified. Volcano plot analysis revealed 13 differentially abundant metabolites for EFA1, 41 for EFA2, and 19 for EFA3 compared to CON group. PLS-DA analysis showed clear separation of treatments, indicating modifications in the rumen fluid metabolome. Conclusions: The treatments led to the enrichment of pathways involved in amino acid, nucleotide, cofactor, and energy metabolism. These additives have the potential to optimize nutrient utilization and overall animal health. Therefore, in vivo studies should be conducted to validate their efficacy.

## 1. Introduction

Improving the production efficiency of dairy cows through optimized feed utilization is a central objective in modern ruminant nutrition research. Enhancing fiber digestion and nutrient conversion not only boosts milk yield but also offers significant economic and environmental benefits. The rumen, as the primary site of microbial fermentation, plays a pivotal role in determining feed efficiency and overall productivity. However, the balance of the ruminal microbiome can be altered by dietary or environmental factors, influencing fermentation efficiency, methanogenesis, and the production of metabolites that regulate host physiology and productivity [1]. Consequently, nutritional interventions aimed at manipulating the rumen environment have become a cornerstone for improving dairy cow productivity while mitigating environmental impacts. Modifying rumen fermentation through dietary strategies such as supplementation with essential oils (EOs), fumaric acid (FA), plant-derived methanogen inhibitors, and alternative hydrogen sinks has emerged as a promising avenue to simultaneously improve performance and reduce greenhouse gas (GHG) emissions [2,3,4].

Essential oils are volatile, plant-derived compounds with antimicrobial, antioxidant, and anti-inflammatory properties. In livestock nutrition, they are considered potential alternatives to antibiotic growth promoters due to their ability to modulate gut microbiota and improve animal performance [5,6,7]. Their application in ruminants has shown promise in reducing methane (CH_4_) emissions, improving feed efficiency, and modulating rumen microbial communities [8,9]. However, research has yielded inconsistent results, with reported variations depending on EO type, dose, animal physiological state, and diet composition [10]. Some studies have documented beneficial effects on nutrient digestibility and milk yield, while others observed transient or negligible effects, highlighting the complexity of EO-microbiome interactions [11,12]. These discrepancies suggest that EOs alone may not consistently produce the desired effects, prompting growing interest in combining multiple essential oils into unique blends or integrating them with other feed additives as innovative feeding strategies.

The chemical diversity of EOs underpins their functional versatility. Combining individual EOs into essential oil blends (EOBs) has been proposed as a strategy to achieve synergistic or additive effects on ruminal fermentation [13,14]. Different EOs contain bioactive molecules such as terpenes, phenolics, and aldehydes, which may target specific microbial populations or metabolic pathways. Thus, EOBs can simultaneously influence multiple fermentation processes, including CH_4_ reduction, volatile fatty acid (VFA) profiles, and microbial protein synthesis. For instance, Blanch et al. [8] reported that supplementation with an EO blend containing cinnamaldehyde and garlic oil reduced CH_4_ emissions and altered VFA patterns in vitro. Similarly, recent findings indicate that combining multiple EOs can improve energy partitioning by increasing propionate production while reducing acetate-to-propionate ratios [15,16]. Given their complementary bioactive properties, EOBs offer a promising approach to optimize rumen fermentation beyond the capacity of individual essential oils. Moreover, the efficacy of EOBs when combined with other anti-methanogenic agents warrants further investigation.

Fumaric acid, an intermediate of the tricarboxylic acid cycle and the succinate–propionate pathway, has attracted attention as a hydrogen sink in the rumen. By redirecting reducing equivalents from methanogenesis to propionate formation, FA supplementation enhances energy capture for the host animal while reducing CH_4_ production [17,18]. FA has been shown to decrease the acetate-to-propionate ratio, reduce total CH_4_ emissions, and improve nutrient utilization efficiency. Importantly, combining FA with EOBs may provide a dual mechanism: while EOs suppress specific microbial groups such as methanogens and protozoa, FA directly competes for hydrogen, further reducing CH_4_ yield. Lin et al. [19] demonstrated that supplementing fumarate (10 mmol/L) along with a blend of essential oils (500 mg/L) containing equal parts of thyme, oregano, cinnamon, and lemon reduced CH_4_ production by 80.2%. This reduction was accompanied by a slight decrease in total VFA and gas production, primarily attributed to the inhibition of protozoa and methanogens. Previous work by Alabi et al. [16] demonstrated that EOB and FA supplementation synergistically reduced CH_4_ and carbon dioxide emissions, while also increasing propionate concentrations and shifting fermentation patterns in favor of energy efficiency. Such interactions highlight the potential of integrated feeding strategies for enhancing rumen function and reducing the environmental footprint of dairy production.

To fully elucidate the mechanisms underpinning such combined effects, advanced analytical approaches are required. Metabolomics, the comprehensive study of small molecules in biological systems, offers a powerful tool to characterize rumen fermentation and nutrient metabolism at the molecular level. By capturing dynamic metabolite profiles, metabolomics provides insights into host–microbe–diet interactions, thereby advancing systems-level understanding of rumen function [20,21]. In ruminant nutrition, metabolomic profiling has been applied to evaluate feed efficiency, energy metabolism, and the impact of dietary interventions on ruminal microbial activity [22]. Metabolomic analyses using liquid chromatography–mass spectrometry (LC–MS) and nuclear magnetic resonance (NMR) spectroscopy have revealed significant associations between diet composition, microbial community shifts, and host metabolic responses [23,24]. Importantly, these high-throughput tools provide a holistic perspective that integrates microbial ecology with host physiology, thereby allowing researchers to trace nutrient fluxes and energy partitioning at unprecedented resolution.

Specific applications of metabolomics in ruminants have demonstrated its utility in linking dietary interventions with metabolic outcomes. Zhang et al. [25] showed that oregano EO supplementation modified ruminal epithelial development and microbiota composition in beef cattle, influencing coenzyme A biosynthesis and pantothenate pathways. Similarly, Li et al. [18] integrated microbiome and metabolome analyses to reveal how EOs shape intestinal metabolite profiles and microbial communities in piglets. In beef cows, Okedoyin et al. [26] reported that supplementation with EOBs containing anise, clove, oregano, and peppermint significantly altered rumen metabolite profiles, including branched-chain VFAs, indicating shifts in microbial fermentation pathways. More recently, Huang et al. [27] found that oregano EO supplementation improved rumen development, digestive efficiency, and growth performance in Holstein steers, while reducing basal energy metabolism, highlighting the broader physiological impacts of EOs. These findings illustrate how metabolomics can uncover subtle biochemical shifts that conventional fermentation measurements may overlook.

Despite these advancements, limited studies have addressed how combinations of EOBs and FA affect the rumen metabolome. Understanding their combined effects could provide crucial mechanistic insights into CH_4_ mitigation and nutrient efficiency strategies. Given the dual roles of EOs in modulating microbial populations and FA as an alternative hydrogen sink, exploring their combined potential through metabolomic approaches is timely and relevant. By identifying metabolic signatures and pathways associated with these interventions, it is possible to optimize feed additive strategies for sustainable dairy production. Therefore, the present study investigated the combined effects of EOBs and fumaric acid on the rumen metabolome using a rumen simulation technique (RUSITEC). This study aimed to provide a comprehensive understanding of how EOB–FA combinations influence metabolite production, microbial fermentation patterns, and key metabolic pathways. The findings are expected to contribute to the development of effective, natural feed additives that enhance nutrient utilization, reduce GHG emissions, and improve the overall productivity and health of dairy cows. In doing so, this research advances the broader agenda of climate-smart animal agriculture and the transition toward sustainable dairy production systems.

## 2. Materials and Methods

### 2.1. The Study Approval

All experimental procedures were approved by the Institutional Animal Care and Use Committee of North Carolina Agricultural and Technical State University, Greensboro (protocol number: LA22-0019), and animals were cared for in accordance with the University Farm standards for animal welfare.

### 2.2. Experimental Design

The total mixed ration (TMR) was formulated to contain corn silage, alfalfa hay, and concentrate at a ratio of 3:1:1 on dry matter (DM) basis. The detailed ingredients and nutrient composition of the TMR have been previously reported [15]. Three unique blends of EOs used in this study were formulated from 11 individual EOs as follows: EOB1 [garlic, lemongrass, cumin, lavender, and nutmeg; ratio 4:2:2:1:1], EOB2 [anise, clove, oregano, cedarwood, and ginger; ratio 4:2:2:1:1], and EOB3 [clove, anise, peppermint, and oregano; ratio 4:3:2:1]. Fumaric acid (≥99% purity) used was procured from Thermo Fisher Scientific (Branchburg, NJ, USA).

The RUSITEC system and the study arrangement have been previously described [15]. Briefly, 16 fermentation chambers (1000 mL capacity each) were randomly allotted to 4 groups with 4 replicates per each group. Each chamber was inoculated with 700 mL of rumen fluid collected from three rumen-cannulated, non-lactating Holstein Friesian cows, along with 200 mL of McDougall’s artificial buffer. The rumen fluid collected from the three cows was pooled and thoroughly mixed before being added to the fermentation chambers to ensure a homogeneous microbial inoculum. The substrate (10 ± 0.2 g) was subjected to 48 h of continuous fermentation under anaerobic conditions. Four dietary treatments were evaluated in a completely randomized design: Control (TMR without additives), EFA1 (TMR + EOB1 + FA), EFA2 (TMR + EOB2 + FA), and EFA3 (TMR + EOB3 + FA). The inclusion dosage of each EOB was 10 µL/g (or 10,000 mg kg^−1^) feed, while FA was added at 3% of TMR DM. Hence, the actual concentration in the rumen fluid phase is approximately 9200 mg kg^−1^ for EOB (assuming the density of the EOB is 0.92 g mL^−1^) and 30,000 mg kg^−1^ for FA. The experimental period lasted 9 d, including a 4-d adaptation phase to allow the microbial community to reach steady-state conditions, followed by 5 days of data collection. The 5-d sampling duration was chosen in accordance with standard RUSITEC protocols, as fermentation parameters and metabolite profiles typically stabilize after the adaptation phase, and longer sampling does not yield additional biological information [28].

### 2.3. Rumen Fluid Sampling and Metabolite Characterization

Rumen fluid samples were collected from the fermenters on days 5, 7, and 9 for metabolomic analysis. Samples (50 mL) from the four replicates within each treatment were pooled into a sterile 250 mL beaker on each sampling day to minimize within-group variability and obtain representative composite samples. From the pooled contents, representative aliquots (45 mL) of liquid effluent were obtained and immediately stored at −80 °C until metabolomic analysis. Thereafter, samples were thawed on ice for approximately 2 h prior to metabolomic analysis. Untargeted metabolome profiling was conducted using chemical isotope labeling (CIL) coupled with liquid chromatography–mass spectrometry (LC–MS). The CIL approach employed 12C/13C-dansylation labeling, which enables group-specific detection of metabolites such as amines/phenols, carboxylic acids, carbonyls (aldehydes and ketones), and hydroxyls. Detailed procedures for sample preparation, isotope labeling, and analytical workflow have been previously described by Idowu et al. [29]. In total, 15 raw LC–MS data files were generated, comprising 12 rumen fluid samples (3 replicates per treatment) and 3 pooled quality control (QC) samples.

Raw LC–MS spectral data were initially exported using Agilent MassHunter software (v12.0, Agilent Technologies, Santa Clara, CA, USA). Data processing and quality assessment were performed with IsoMS Pro (v1.2.16), including peak picking, peak pairing, and peak-pair filtering to eliminate redundant ion peaks (adducts, dimers, and multimers). Metabolite features were identified based on retention time and mass-to-charge ratio (*m*/*z*) by matching against the CIL and linked identity libraries, thereby ensuring high confidence in metabolite identification [29]. To ensure analytical reliability, features with a relative standard deviation (RSD) ≥ 25% across pooled quality control (QC) samples were removed, resulting in a median QC coefficient of variation (CV) of 11.8% among retained metabolites.

### 2.4. Untargeted Metabolome Analysis

Processed metabolomic data were uploaded to the MetaboAnalyst 6.0 platform (https://www.metaboanalyst.ca/ (accessed on 15 August 2025)) for statistical and pathway analyses. Prior to multivariate modeling, data integrity checks were performed, followed by normalization using median-centering, log transformation, and autoscaling, as recommended for high-dimensional metabolomics datasets [30]. Chemometric analysis was performed using Partial Least Squares Discriminant Analysis (PLS-DA) to visualize separation between treatment and control groups based on metabolite profiles. Model robustness was evaluated through permutation testing (*n* = 100) to validate the significance of R^2^Y (Goodness of Fit) and Q^2^ (Predictive Ability). Differentially abundant metabolites were identified using volcano plot analysis, with significance threshold of *p* ≤ 0.05 and an absolute fold-change (FC) ≥ 1.5. To control for false positives arising from multiple comparisons, the Benjamini–Hochberg False Discovery Rate (FDR) correction was applied, and adjusted *q*-values were reported. To evaluate the predictive accuracy and reliability of the PLS-DA model, 5-fold cross-validation was performed using four latent components. Model metrics including accuracy, R^2^, and Q^2^ values were calculated to assess model stability and predictive power during cross-validation.

Receiver Operating Characteristic (ROC) curve analysis was then employed to evaluate the discriminatory power of individual metabolites. Candidates’ biomarkers were identified based on their area under the curve (AUC) values, with AUC values approaching 1.0 indicating higher predictive accuracy [31]. Finally, pathway enrichment analysis was conducted using the Kyoto Encyclopedia of Genes and Genomes (KEGG) database to identify metabolic pathways significantly affected by dietary treatments (EFA1, EFA2, and EFA3) relative to the control. Enrichment was considered significant at *p* ≤ 0.05, thereby providing insights into the metabolic and biochemical pathways influenced by supplementation with EOBs and FA. For each pathway, the number of matched metabolites (Hits), total pathway size, the enrichment ratio (Hits/Total), raw *p*-value and FDR-adjusted *q*-value (Benjamini–Hochberg) were reported.

## 3. Results

The boxplot of samples of the metabolome between the treated and control group before- and after- normalization is shown in Figure S1. A total of 661 distinct metabolites were successfully detected and annotated (see Supplementary Tables). Partial Least Squares Discriminant Analysis (PLS-DA) revealed a clear separation between the treatment and control groups, with the majority of the discriminatory variation by explained Components 1 and 2. This finding suggests that the treatment altered a specific subset of metabolites. The permutation test confirmed robustness of the PLS-DA model, with significant empirical *p*-values for both Q^2^ (*p* ≤ 0.01) and R^2^Y (*p* ≤ 0.01), demonstrating that the observed group separation was highly unlikely to have occurred by random chance (Figure S2). These results provide strong evidence that supplementation with EOs and FA produced significant metabolic alterations relative to the control group, with key discriminatory metabolites identifiable through Variable Importance in Projection (VIP) analysis.

**Figure 1:**
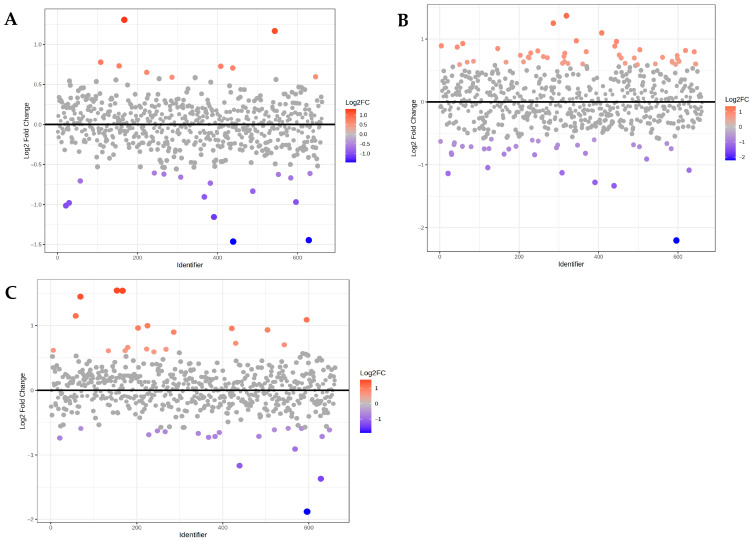
Fold change plot of metabolites comparing (**A**) EFA1 and CON group; (**B**) EFA2 and CON group; and (**C**) EFA3 and CON group.

The fold-change plot analysis of the EFA1 group showed that 16 metabolites were significantly downregulated (*p* < 0.05) and 9 were upregulated compared to the CON group (Figure 1A; Table 1). In the EFA2 group, 33 metabolites were significantly downregulated (*p* < 0.05) and 47 were upregulated compared to the CON group (Figure 1B; Table 2). For the EFA3 group, 19 metabolites were significantly downregulated and 19 were upregulated compared to the CON group (Figure 1C; Table 3).

**Figure 2:**
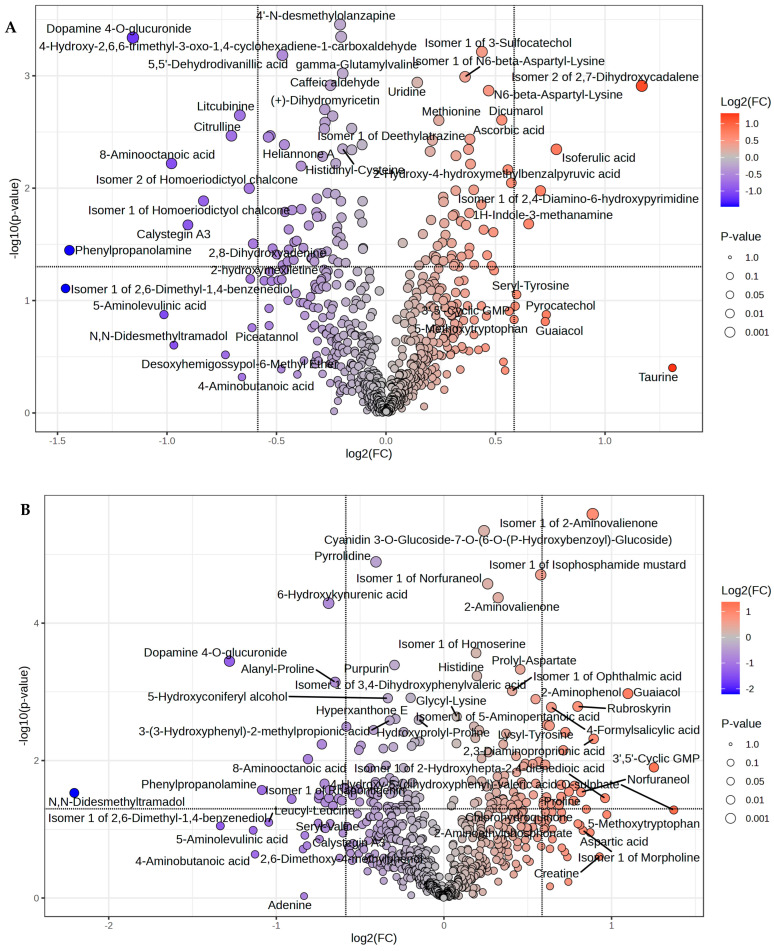

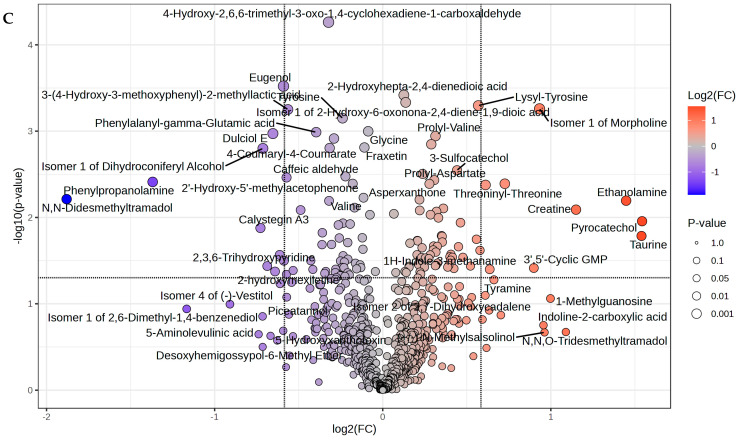
Volcano plot showing the differential rumen metabolites between (**A**) CON and EFA1 group; (**B**) CON and EFA2 group; and (**C**) CON and EFA3 group.

Volcano plot combines results from Fold Change (FC) Analysis and *T*-tests into a single graph, allowing the intuitive selection of significant features based on biological significance, statistical significance, or both. The volcano plot showed that a total of 13 differentially abundant (FC ≥ 1.5, *p* ≤ 0.05) metabolites were detected between EFA1 and CON. Compared to the CON, 4 metabolites were significantly (*p* < 0.05) upregulated while 9 were downregulated in EFA1 group (Figure 2A). Isomer 2 of 2,7-Dihydroxycadalene showed a 2.25-fold increase, Isoferulic acid a 1.71-fold increase, and Isomer 1 of 2,4-Diamino-6-hydroxypyrimidine a 1.63-fold increase. In contrast, Phenylpropanolamine experienced a 0.37-fold decrease, Dopamine 4-*O*-glucuronide a 0.45-fold decrease, and 8-Aminooctanoic acid a 0.51-fold decrease.

In EFA2 group, a total of 41 differentially abundant (FC ≥ 1.5, *p* ≤ 0.05) metabolites were detected, 23 metabolites were differentially (*p* < 0.05) upregulated while 18 were downregulated compared to the CON group (Figure 2B). The volcano plot revealed that 3′,5′-Cyclic GMP exhibited a 2.38-fold increase, Guaiacol a 2.14-fold increase, Isomer 1 of 2-Hydroxyhepta-2,4-dienedioic acid a 1.95-fold increase, 2,3-Diaminopropionic acid a 1.86-fold increase, and Isomer 1 of 2-Aminovalienone a 1.85-fold increase. In contrast, *N*,*N*-Didesmethyltramadol showed a 0.22-fold decrease, Dopamine 4-*O*-glucuronide a 0.41-fold decrease, Phenylpropanolamine a 0.47-fold decrease, Isomer 1 of Rhapontigenin a 0.53-fold decrease, and 8-Aminooctanoic acid a 0.57-fold decrease.

In EFA3 group, volcano plot showed that a total of 19 differentially abundant (FC ≥ 1.5, *p* ≤ 0.05) metabolites were detected, 9 metabolites were significantly (*p* < 0.05) higher while 10 were lower compared to the CON group (Figure 2C). Pyrocatechol exhibited a 2.91-fold increase, Taurine a 2.90-fold increase, Ethanolamine a 2.73-fold increase, Creatine a 2.22-fold increase, Isomer 1 of Morpholine a 1.91-fold increase, and 3′,5′-Cyclic GMP a 1.86-fold increase. In contrast, Isomer 1 of Dihydroconiferyl Alcohol showed a 0.61-fold decrease, Calystegin A3 a 0.60-fold decrease, Phenylpropanolamine a 0.39-fold decrease, and *N*,*N*-Didesmethyltramadol a 0.27-fold decrease.

As outlined in Table 4, all EFA groups demonstrated perfect classification accuracy (accuracy = 1.000). The R^2^ values ranged from 0.9988 to 0.9998 for EFA1, 0.9676 to 0.9998 for EFA2, and 0.9944 to 0.9989 for EFA3, indicating excellent model fit across all groups. Corresponding Q^2^ values, reflecting predictive ability, ranged from 0.5657 to 0.9925 for EFA1, 0.7105 to 0.9902 for EFA2, and 0.5984 to 0.9884 for EFA3, further supporting the robustness and reliability of the models.

**Figure 3:**
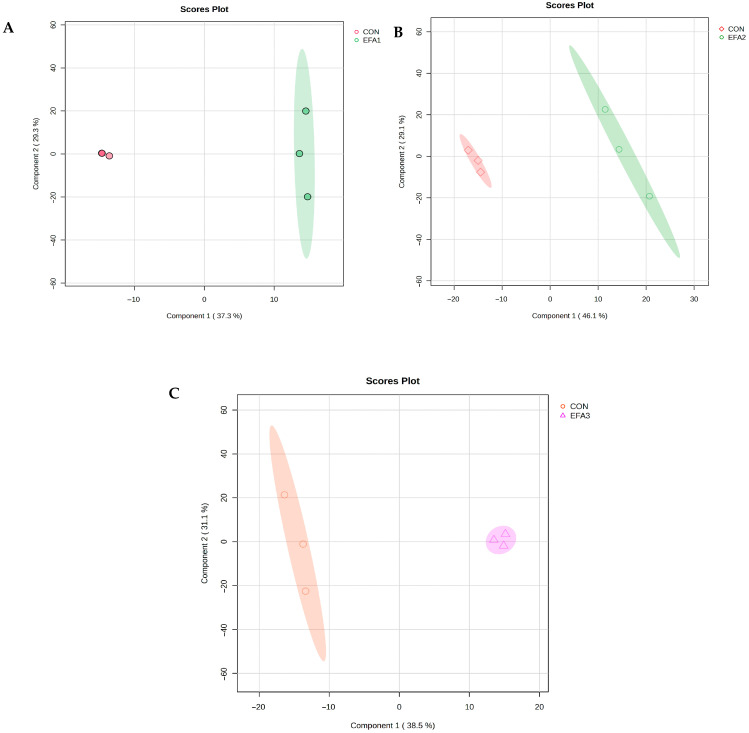
PLS-DA scores plot showing the metabolome between (**A**) CON and EFA1 group; (**B**) CON and EFA2 group; and (**C**) CON and EFA3 group.

The PLS-DA scores plot demonstrated clear separation between the two treatment groups based on the first two principal components, with explained variances of 37.3 and 29.3% for EFA1 (Figure 3A), 46.1 and 29.1% for EFA2 (Figure 3B), and 38.5 and 31.1% for EFA3 (Figure 3C), suggesting that feed additives modified the rumen fluid metabolome.

**Figure 4:**
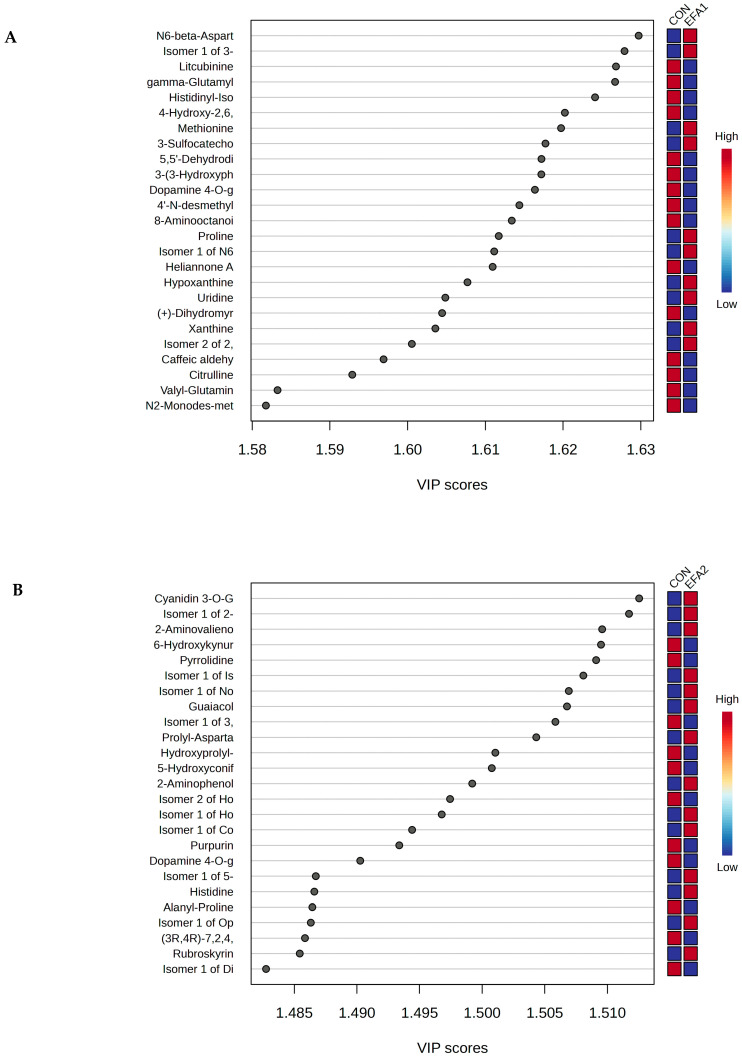

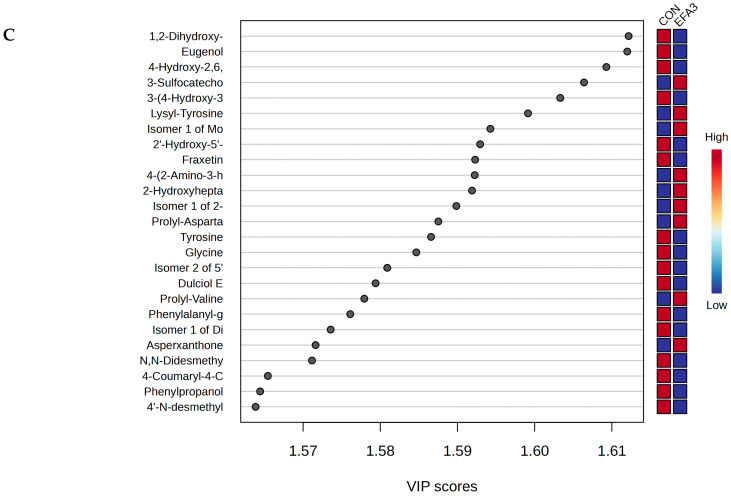
PLS-DA VIP scores show the metabolome between (**A**) CON and EFA1 group; (**B**) CON and EFA2 group; and (**C**) CON and EFA3 group.

As shown in Figure 4A, the PLS-DA VIP Scores revealed that EFA1 increased the concentration of *N*6-beta-Aspartyl-Lysine, Isomer 1 of 3-Sulfocatechol, Methionine, 3-Sulfocatechol, Proline, Isomer 1 of *N*6-beta-Aspartyl-Lysine, Hypoxanthine, Uridine, Xanthine and Isomer 2 of 2,7-Dihydroxycadalene whereas the concentration of Litcubinine, gamma-Glutamylvaline, Histidinyl-Isoleucine, 4-Hydroxy-2,6,6-trimethyl-3-oxo-1,4-cyclohexadiene-1-carboxaldehyde, 5,5′-Dehydrodivanillic acid, 3-(3-Hydroxyphenyl)-2-methylpropionic acid, Dopamine 4-*O*-glucuronide, 4′-*N*-desmethylolanzapine, 8-Aminooctanoic acid, Heliannone A and Citrulline were higher in CON group.

The PLS-DA VIP Scores plot (Figure 4B) revealed that EFA2 increased the concentration of Cyanidin 3-*O*-Glucoside-7-*O*-(6-*O*-(P-Hydroxybenzoyl)-Glucoside), Isomer 1 of 2-Aminovalienone, 2-Aminovalienone, Isomer 1 of Isophosphamide mustard, Isomer 1 of Norfuraneol, Guaiacol, Prolyl-Aspartate, 2-Aminophenol, Isomer 1 of Homoserine, Isomer 1 of Coniferyl aldehyde, Isomer 1 of 5-Aminopentanoic acid, Histidine, Isomer 1 of Ophthalmic acid and Rubroskyrin. However, 6-Hydroxykynurenic acid, Pyrrolidine, Isomer 1 of 3,4-Dihydroxyphenylvaleric acid, Hydroxyprolyl-Proline, 5-Hydroxyconiferyl alcohol, Isomer 2 of Homoeriodictyol chalcone, Purpurin, Dopamine 4-*O*-glucuronide, Alanyl-Proline, (3*R*,4*R*)-7,2,4,2′-Tetrahydroxy-4′,5′-Methylenedioxyisoflav-3-Ene, and Isomer 1 of Dihydroconiferyl Alcohol were higher in CON group. The PLS-DA VIP Scores plot (Figure 4C) revealed that EFA3 increased the concentration of 3-Sulfocatechol, Lysyl-Tyrosine, Isomer 1 of Morpholine, 4-(2-Amino-3-hydroxyphenyl)-2,4-dioxobutanoic acid, 2-Hydroxyhepta-2,4-dienedioic acid, Isomer 1 of 2-Hydroxy-6-oxonona-2,4-diene-1,9-dioic acid, Prolyl-Aspartate, Prolyl-Valine and Asperxanthone. Meanwhile, the concentration of 1,2-Dihydroxy-8-methylnaphthalene, Eugenol, 4-Hydroxy-2,6,6-trimethyl-3-oxo-1,4-cyclohexadiene-1-carboxaldehyde, 3-(4-Hydroxy-3-methoxyphenyl)-2-methyllactic acid, 2′-Hydroxy-5′-methylacetophenone, Fraxetin, Tyrosine, Glycine, Isomer 2 of 5′-(3′-Methoxy-4′-hydroxyphenyl)-gamma-valerolactone, Dulciol E, Phenylalanyl-gamma-Glutamic acid, Isomer 1 of Dihydroconiferyl Alcohol, *N*,*N*-Didesmethyltramadol, 4-Coumaryl-4-Coumarate, Phenylpropanolamine, and 4′-*N*-desmethylolanzapine were higher in CON group.

**Figure 5:**
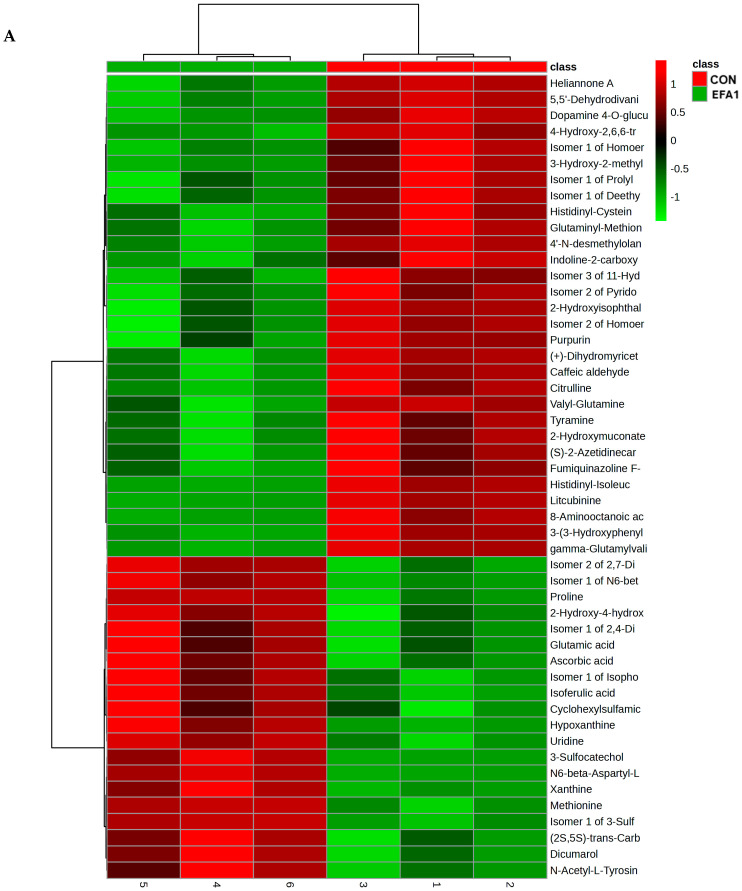

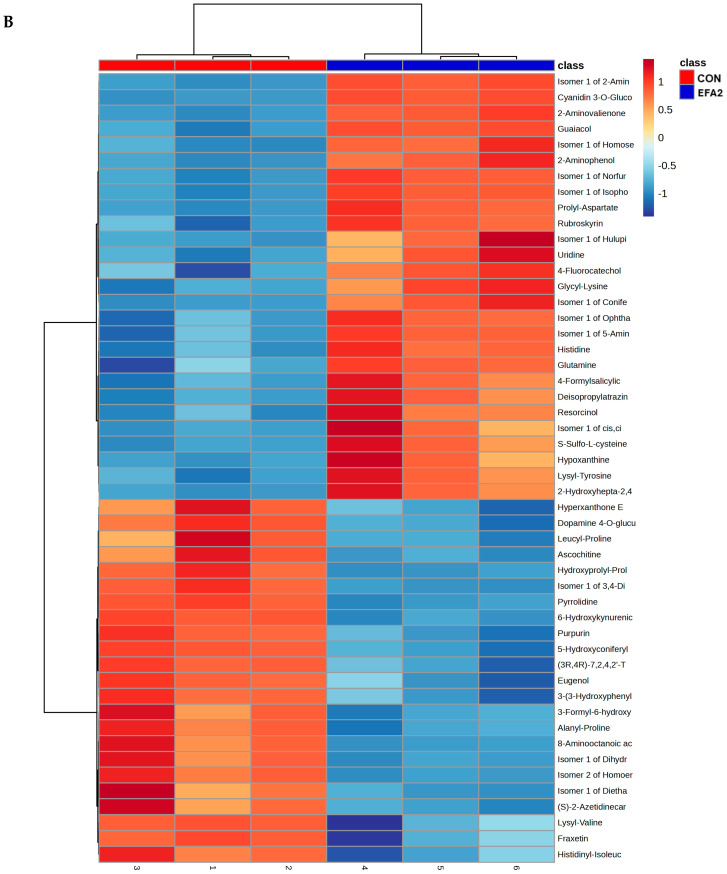

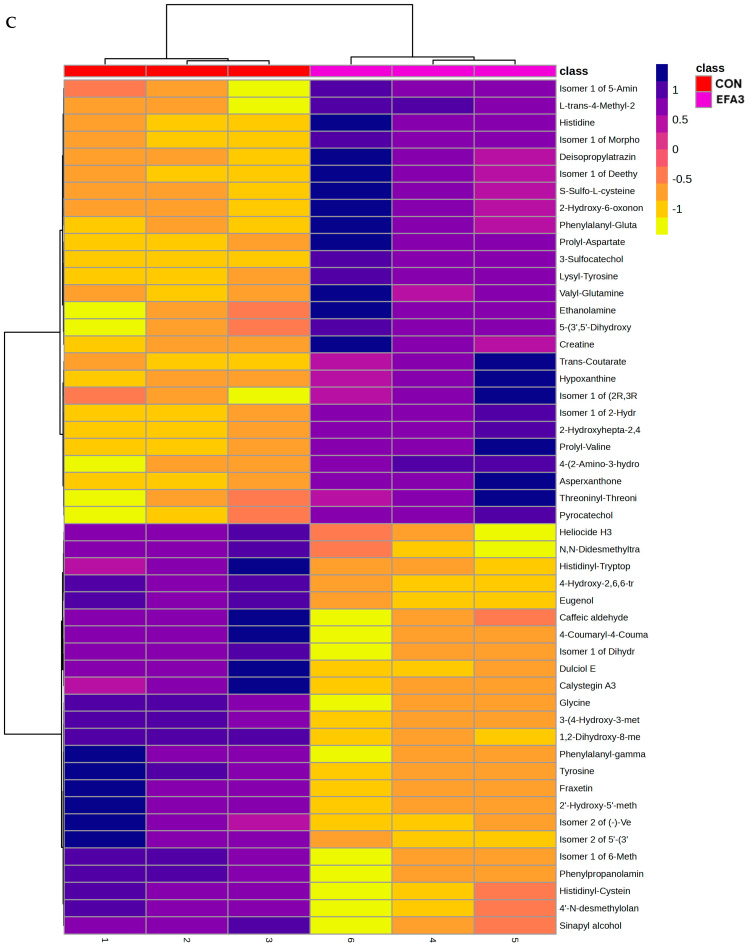
Heatmap showing the 50 top differential rumen metabolites between (**A**) CON and EFA1 group; (**B**) CON and EFA2 group; and (**C**) CON and EFA3 group.

The heatmap of the 50 top differentially abundant metabolites confirmed that EFA1 increased the abundance of *N*6-beta-Aspartyl-Lysine, Isomer 1 of 3-Sulfocatechol, Methionine, 3-Sulfocatechol, Proline, Isomer 1 of *N*6-beta-Aspartyl-Lysine, Hypoxanthine, Uridine, Xanthine and Isomer 2 of 2,7-Dihydroxycadalene, Isomer 2 of 2,7-Dihydroxycadalene, Isomer 1 of *N*6-beta-Aspartyl-Lysine, Proline, 2-Hydroxy-4-hydroxymethylbenzalpyruvic acid, Isomer 1 of 2,4-Diamino-6-hydroxypyrimidine, Glutamic acid, Ascorbic acid, Isomer 1 of Isophosphamide mustard, Isoferulic acid, Cyclohexylsulfamic acid, and Dicumarol (Figure 5A).

Figure 5B confirmed that EFA2 increased the concentration of Cyanidin 3-*O*-Glucoside-7-*O*-(6-*O*-(*P*-Hydroxybenzoyl)-Glucoside), Isomer 1 of 2-Aminovalienone, 2-Aminovalienone, Isomer 1 of Isophosphamide mustard, Isomer 1 of Norfuraneol, Guaiacol, Prolyl-Aspartate, 2-Aminophenol, Isomer 1 of Homoserine, Isomer 1 of Coniferyl aldehyde, Isomer 1 of 5-Aminopentanoic acid, Isomer 1 of Ophthalmic acid, Rubroskyrin, *S*-Sulfo-l-cysteine, Resorcinol, Histidine, Glutamine, 4-Formylsalicylic acid, Glycyl-Lysine, 4-Fluorocatechol, and 2-Hydroxyhepta-2,4-dienedioic acid.

Figure 5C revealed that EFA3 increased the concentration of Isomer 1 of 5-Aminopentanoic acid, l-*trans*-4-Methyl-2-pyrrolidinecarboxylic acid, Isomer 1 of Morpholine, Deisopropylatrazine, Isomer 1 of Deethylatrazine, 3-Sulfocatechol, Lysyl-Tyrosine, 4-(2-Amino-3-hydroxyphenyl)-2,4-dioxobutanoic acid, 2-Hydroxyhepta-2,4-dienedioic acid, Isomer 1 of 2-Hydroxy-6-oxonona-2,4-diene-1,9-dioic acid, Prolyl-Aspartate, Prolyl-Valine, 4-(2-Amino-3-hydroxyphenyl)-2,4-dioxobutanoic acid and Asperxanthone.

**Figure 6:**
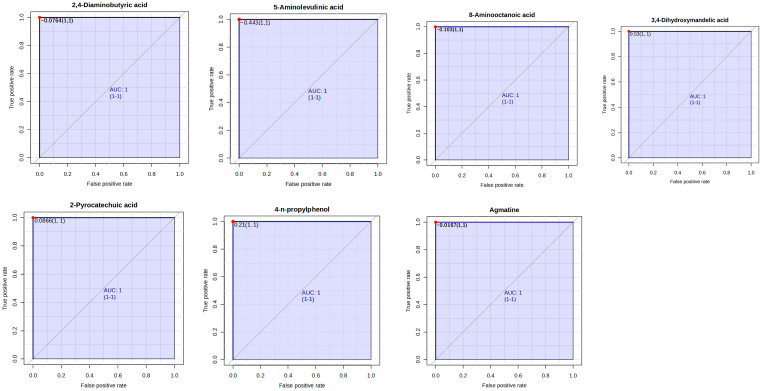
Receiver–operator characteristic curves of 2,4-Diaminobutyric acid, 5-Aminolevulinic acid, 8-Aminooctanoic acid, 3,4-Dihydroxymandelic acid, 2-Pyrocatechuic acid, 4-n-propylphenol, and Agmatine which are biomarkers enhanced by EFA1.

**Figure 7:**
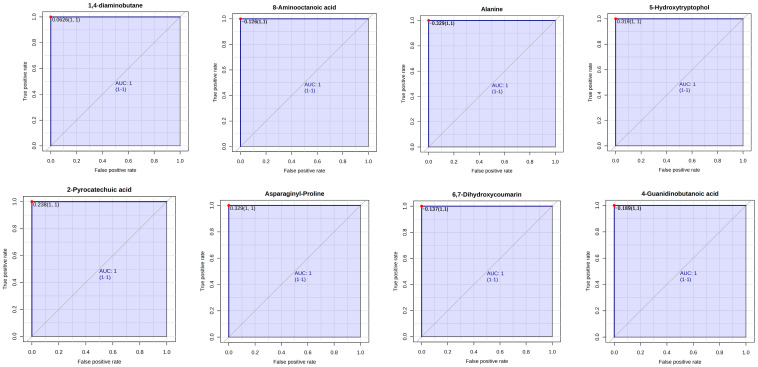
Receiver–operator characteristic curves of 1,4-diaminobutane, 8-Aminooctanoic acid, Alanine, 5-Hydroxytryptophol, 2-Pyrocatechuic acid, Asparaginyl-Proline, 6,7-Dihydroxycoumarin, and 4-Guanidinobutanoic acid which are biomarkers enhanced by EFA2.

**Figure 8:**
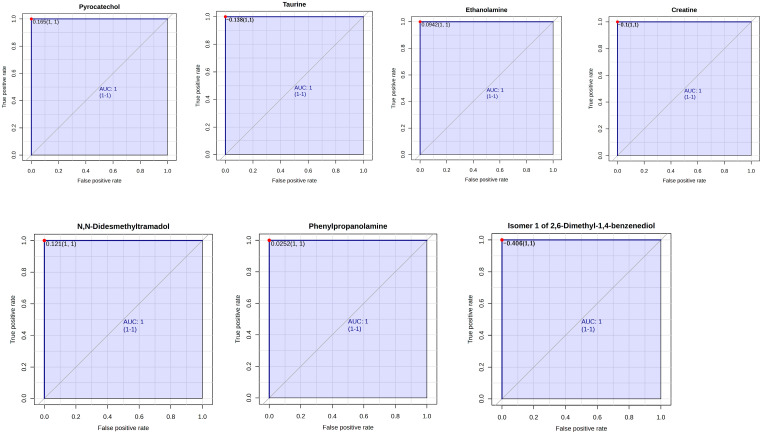
Receiver–operator characteristic curves of Pyrocatechol, Taurine, Ethanolamine, Creatine, *N*,*N*-Didesmethyltramadol, Phenylpropanolamine, and Isomer 1 of 2,6-Dimethyl-1,4-benzenediol which are biomarkers enhanced by EFA3.

The ruminal metabolites which served as biomarkers were assessed using Receiver–operator characteristic (ROC) curves. According to the accepted classification of biomarker utility, candidate markers of AUC greater than or equal to 0.9 are considered “excellent”. In this study, rumen metabolites that are key biomarkers (AUC = 1) enhanced by EFA1 include 2,4-Diaminobutyric acid, 5-Aminolevulinic acid, 8-Aminooctanoic acid, 3,4-Dihydroxymandelic acid, 2-Pyrocatechuic acid, 4-n-propylphenol, and Agmatine (Figure 6). Biomarkers enhanced by EFA2 include 1,4-diaminobutane, 8-Aminooctanoic acid, Alanine, 2-Pyrocatechuic acid, 5-Hydroxytryptophol, Asparaginyl-Proline, 6,7-Dihydroxycoumarin, and 4-Guanidinobutanoic acid (Figure 7). Notable biomarkers enhanced by EFA3 include Pyrocatechol, Taurine, Ethanolamine, Creatine, *N*,*N*-Didesmethyltramadol, Phenylpropanolamine, and Isomer 1 of 2,6-Dimethyl-1,4-benzenediol (Figure 8).

**Figure 9:**
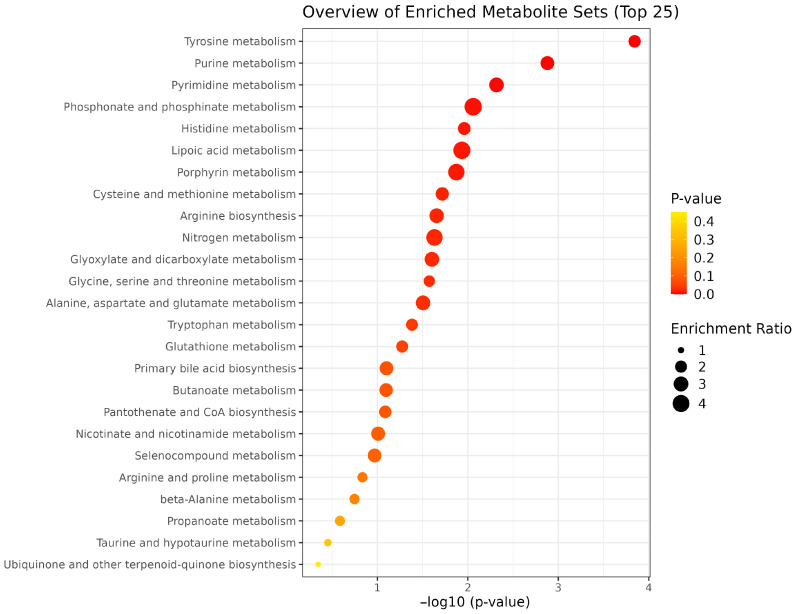
Enrichment pathway analysis by EFA1 and CON group.

The enrichment pathway analysis revealed that EFA1 treatment led to the enrichment of tyrosine, purine, pyrimidine, lipoic acid, porphyrin, and phosphate metabolism pathways (Figure 9). Tyrosine metabolism exhibited the highest statistical significance (FDR = 5.7 × 10^−6^; Impact = 0.310), followed by purine metabolism (FDR = 1.1 × 10^−4^; Impact = 0.157) and pyrimidine metabolism (FDR = 5.8 × 10^−4^; Impact = 0.154). Meanwhile, arginine biosynthesis (Impact = 0.429) and histidine metabolism (Impact = 0.375) demonstrated the highest pathway coverage (Table 5).

**Figure 10:**
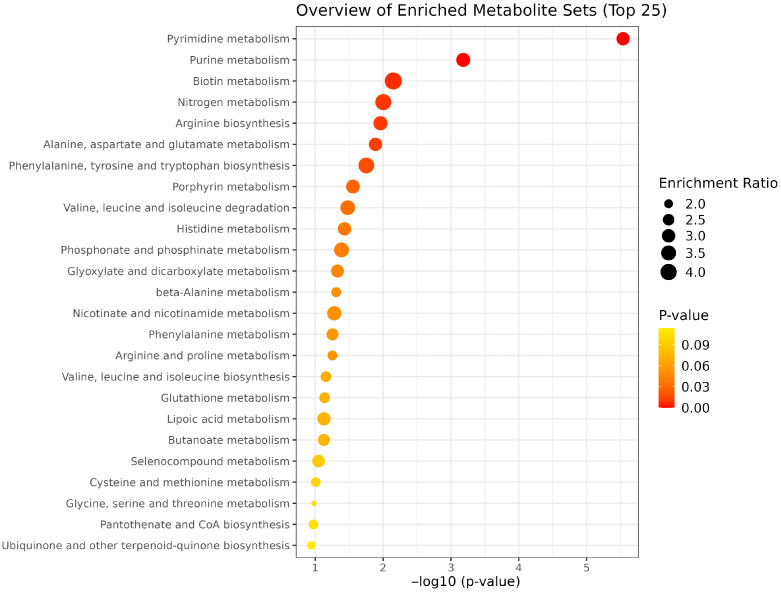
Enrichment pathway analysis by EFA2 and CON group.

**Figure 11:**
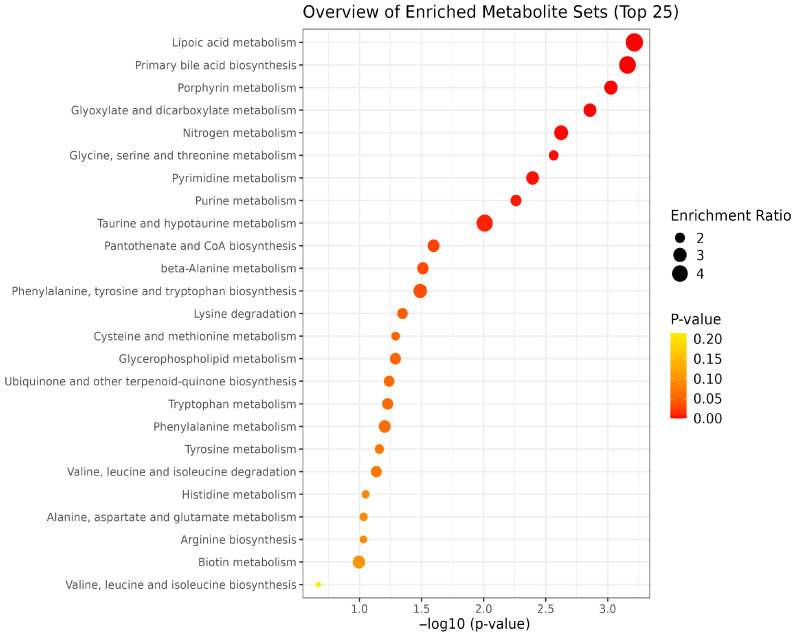
Enrichment pathway analysis of differential rumen metabolites by EFA3 and CON group.

EFA2 supplementation enriched key metabolic pathways, including pyrimidine, purine, biotin, and nitrogen metabolism, as well as the metabolism of alanine, aspartate, and glutamate, and the biosynthesis of arginine, phenylalanine, tyrosine, and tryptophan (Figure 10). Among the pathways altered by EFA2 treatment, pyrimidine metabolism and purine metabolism showed the lowest *p*- and FDR-values (Table 6). High impact values were observed in phenylalanine, tyrosine and tryptophan biosynthesis (0.5) and arginine biosynthesis (0.429) whereas moderate impact (0.2–0.4) were observed in alanine, aspartate and glutamate metabolism and histidine metabolism. EFA3 supplementation resulted in the enrichment of multiple metabolic pathways, including those related to lipoic acid, porphyrin, glyoxylate and dicarboxylate metabolism, nitrogen cycling, glycine, serine, and threonine metabolism, taurine and hypotaurine metabolism, and primary bile acid biosynthesis (Figure 11). Porphyrin metabolism, lipoic acid metabolism, and primary bile acid biosynthesis exhibited the lowest *p*- and FDR-values, indicating strong statistical significance (Table 7). High enrichment impacts were observed in phenylalanine, tyrosine and tryptophan biosynthesis (0.50), arginine biosynthesis (0.429), and phenylalanine metabolism (0.375).

## 4. Discussion

The supplementation of different EOBs combined with fumaric acid (EFA1, EFA2, and EFA3) resulted in distinct metabolite profiles that indicated specific alterations in rumen fermentation pathways linked to methanogenesis and overall rumen function. The EFA1 treatment, containing garlic, lemongrass, cumin, lavender, and nutmeg with fumaric acid, altered several key metabolites associated with rumen microbial activity. The significant upregulation of 2,7-dihydroxycadalene (2.25-fold) is particularly noteworthy, as cadalene derivatives have been reported to exhibit antimicrobial properties that may selectively inhibit methanogenic archaea [13]. Isoferulic acid upregulation (1.71-fold) suggests enhanced phenylpropanoid metabolism, which has been linked to reduced CH_4_ production through inhibition of hydrogen-producing bacteria [32]. This compound, commonly found in plant sources, possesses antioxidant properties that may improve rumen health while modulating fermentation patterns [33].

The downregulation of phenylpropanolamine (0.37-fold) and dopamine 4-*O*-glucuronide (0.45-fold) indicates altered catecholamine metabolism. These changes may reflect shifts in microbial populations, particularly those involved in aromatic amino acid degradation pathways [9]. The observed 0.51-fold decrease in 8-aminooctanoic acid (8-aminocaprylic acid) suggests shifts in fatty acid metabolism that may indirectly influence methanogenesis [34]. This compound, known as an intermediate in amino–fatty acid catabolism and a modulator of aminergic/GABAergic pathways, can originate from microbial degradation of dietary lipids or atypical amino acid metabolism. In the rumen, such medium-chain amino–fatty acids may disrupt microbial membrane integrity, altering permeability and protein function, thereby potentially inhibiting microbial growth or shifting population dynamics [35].

The garlic component of EOB1 in EFA1 likely contributed organosulfur compounds that inhibit the activity of HMG-CoA reductase in methanogens, thereby disrupting cell membrane synthesis [36]. Additionally, lemongrass bioactives, primarily citral, may have enhanced propionate production as earlier reported [15,16], creating an alternative hydrogen sink that competes with methanogenesis [37]. The observed upregulation of specific metabolites in the EFA1 treatment reveals significant alterations in several metabolic pathways with important implications for dairy cow health, productivity, and methanogenesis. The increased concentration of *N*6-beta-Aspartyl-Lysine and its isomer suggests enhanced protein turnover and dipeptide metabolism in the rumen. These dipeptides are intermediates in protein degradation pathways and their elevation indicates more efficient proteolysis by rumen microorganisms [38]. This could lead to improved microbial protein synthesis and potentially better nitrogen utilization efficiency. Enhanced microbial protein synthesis is particularly beneficial for dairy cows, as it contributes significantly to the metabolizable protein reaching the small intestine, supporting milk protein synthesis [39].

Metabolomic shifts in the EFA1 treatment reveal tighter coupling of ruminal nitrogen, energy and redox pathways. First, the elevation in methionine is particularly important because this sulfur-containing essential amino acid is frequently limiting in dairy rations. Higher ruminal methionine supply supports milk-protein synthesis, hepatic very-low-density-lipoprotein (VLDL) assembly for lipid export, and immune and antioxidant defense through its conversion to cysteine and glutathione [40]. Methionine-derived intermediates may also serve as alternative hydrogen sinks, diverting reducing equivalents away from methanogenesis and thereby contributing to lower CH_4_ output [41]. Second, the increase in proline signals complementary shifts in amino acid metabolism. Proline functions as a substrate for hepatic gluconeogenesis, a structural component of milk proteins, and a precursor for ornithine in the urea cycle. Elevated proline therefore suggests more efficient nitrogen recycling within the rumen and improved host *N* utilization, which can reduce urinary N losses without compromising milk yield [42]. It also supports collagen turnover and osmotic balance, helping to preserve rumen-epithelial integrity under dietary stress. Higher methionine and proline levels denote more efficient amino-acid assimilation and microbial protein synthesis [31], underpinning both improved production performance and reduced ruminal ammonia accumulation, which are critical steps toward lowering downstream nitrous oxide emissions. These metabolic improvements reinforce antioxidant defenses [43] and bolster the rumen epithelium, facilitating the cow’s physiological adaptation to dietary change [44].

The upregulation of hypoxanthine, xanthine, and uridine observed in the EFA1 treatment group suggests a substantial shift in nucleotide metabolism, with important implications for rumen microbial activity, energy dynamics, and nitrogen utilization. Hypoxanthine and xanthine are intermediates in the purine degradation pathway that culminates in uric acid formation via xanthine oxidoreductase activity [45]. Their elevated concentrations serve as indicators of intensified ATP and GTP turnover, reflecting increased energy demands and cellular activity. These purine derivatives are also widely recognized as reliable markers of microbial protein synthesis in ruminants [46], and their upregulation likely signals enhanced microbial growth and nucleic acid turnover, which can improve the host animal’s protein supply [39].

Similarly, the elevation of uridine, a pyrimidine nucleoside, provides further evidence of active nucleotide metabolism. Uridine contributes to RNA synthesis and membrane phospholipid formation, indicating improved cellular biosynthetic capacity [45]. It can also be metabolized to generate ribose for the pentose phosphate pathway and glycolysis, thereby supporting energy production and redox balance [30]. These changes suggest that the EFA1 treatment enhanced the metabolic activity of the rumen microbiota, promoting more efficient fermentation and microbial protein synthesis [18]. Moreover, increased nucleotide turnover may reflect a microbial community with higher proliferation rates and greater capacity for nitrogen assimilation via salvage pathways, thereby improving nitrogen recycling efficiency. This could lead to a reduction in ruminal ammonia accumulation and a decrease in nitrogenous emissions, such as nitrous oxide, benefiting both animal productivity and environmental sustainability.

Elevated concentrations of 3-sulfocatechol, its structural isomer, and 2,7-dihydroxycadalene in the EFA1 treatment demonstrate vigorous microbial biotransformation of plant-derived phenolics supplied by the essential-oil blend [47]. Such sulfated and hydroxylated derivatives arise from phase I oxidation followed by phase II conjugation, signaling an efficient detoxification network that augments the rumen’s metabolic resilience [26]. Sulfocatechols, in particular, contribute substantial antioxidant capacity, thereby improving the redox status of the rumen and shielding epithelial tissues from oxidative injury while simultaneously favoring nutrient absorption [48].

Beyond redox effects, these phenolics possess selective antimicrobial activity. The 2.25-fold rise in 2,7-dihydroxycadalene is noteworthy because this cadalene-type sesquiterpenoid disrupts archaeal membranes and key methanogenic enzymes, offering a plausible mechanism for the anti-methanogenic action observed with essential-oil supplementation [9,16]. By curtailing hydrogenotrophic archaea, this compound may divert reducing equivalents toward more energetically favorable fermentation pathways, complementing the hydrogen-sparing role of fumaric acid incorporated in the EFA1 blend. Concurrently, the accumulation of the dipeptide *N*6-β-aspartyl-lysine and related isomers indicates accelerated protein turnover and cross-link repair within the rumen. Higher dipeptide abundance implies both enhanced microbial proteolysis and more efficient nitrogen recycling, consistent with the broader increase in amino-acid availability detected under EFA1. Because metabolomic extraction was performed on ruminal effluent rather than feed residues in the nylon bags, the detected dipeptides are most likely of microbial origin. The rapid degradation of dietary peptides during the incubation period, along with the specific accumulation of *N*6-β-aspartyl-lysine; a metabolite typically linked to microbial protein repair and turnover, further supports this interpretation. Although distinguishing microbial- from feed-derived sources would require isotopic labeling, the observed metabolite profiles under EFA1 align with microbial metabolic responses rather than residual feed effects.

The enrichment of tyrosine, purine, pyrimidine, lipoic acid, porphyrin, and phosphate metabolism under EFA1 treatment reflects key metabolite shifts. Elevated hypoxanthine, xanthine, and uridine suggest enhanced purine and pyrimidine turnover, supporting microbial growth and energy metabolism [30,45]. Increased tyrosine may indicate improved aromatic amino acid metabolism and protein synthesis. Upregulated lipoic acid-related metabolites could enhance mitochondrial redox balance and energy production [49]. Changes in porphyrin metabolism may relate to microbial heme synthesis. Lastly, phosphate and phosphinate pathway shifts may reflect increased nucleotide and phospholipid synthesis essential for microbial proliferation [18,31].

The biological significance of the metabolite profile in the EFA2 group compared to the control group reveals substantial impacts on key metabolic pathways with important implications for dairy cow health and productivity. The substantial increase in 3′,5′-cyclic GMP (2.38-fold) suggests enhanced signal transduction processes that may influence microbial communication and population dynamics. Elevated cGMP levels may modulate the synthesis and detection of quorum sensing molecules, which are essential for coordinating bacterial behavior within communities. These changes in signaling pathways can subsequently affect key collective processes such as biofilm formation, motility, and dispersal [50,51]. Additionally, the increase in 3′,5′-Cyclic GMP suggests enhanced intracellular signaling mechanisms that regulate numerous physiological processes including smooth muscle relaxation and potentially improved blood flow to the rumen epithelium and mammary tissue [52]. Guaiacol upregulation (2.14-fold) is significant as this phenolic compound, derived from lignin degradation, has been shown to inhibit methanogen activity by disrupting membrane integrity [53]. This marked increase suggests shifts in phenolic metabolism with potential antimicrobial and antioxidant properties that may selectively modify rumen fermentation patterns and protect epithelial tissues from oxidative damage [33].

The increase in 2-hydroxyhepta-2,4-dienedioic acid (1.95-fold) and 2,3-diaminopropionic acid (1.86-fold) point to reconfigured carboxylic- and amino-acid metabolic routes. Because these pathways feed directly into microbial protein synthesis, their up-regulation may signal more efficient ruminal nitrogen capture [54]. Redirecting reducing equivalents toward organic-acid formation rather than methanogenesis could also mitigate hydrogen accumulation. Specifically, greater abundance of 2-hydroxyhepta-2,4-dienedioic acid, an intermediate in unsaturated dicarboxylic-acid metabolism, may help stabilize rumen pH and enhance fiber degradation via altered microbial cross-feeding [55]. Likewise, higher levels of 2,3-diaminopropionic acid, a non-proteinogenic amino acid formed during lysine catabolism, may underpin more efficient incorporation of nitrogen into microbial biomass, ultimately increasing the metabolizable protein supplied to the host animal [38].

The pronounced downregulation of *N*,*N*-didesmethyltramadol (0.22-fold) and phenylpropanolamine (0.47-fold) indicates a shift in alkaloid–xenobiotic metabolism, likely driven by the eugenol present in clove oil, which disrupts microbial cytoplasmic membranes [32]. A parallel 0.41-fold decrease in dopamine 4-*O*-glucuronide reinforces the idea of altered aromatic-compound turnover, pointing to modified detoxification pathways within the rumen or host tissues [56]. Lower dopamine conjugates and phenylpropanolamine concentrations suggest changes in aromatic amine metabolism rather than direct modulation of sympathetic activity within the rumen [57]. Because phenylpropanolamine is a sympathomimetic amine structurally related to catecholamines, its concurrent reduction with dopamine conjugates may reflect changes in microbial or host biotransformation and detoxification of bioactive amines, potentially influencing systemic metabolic signaling linked to rumen motility and passage rate. A 0.53-fold reduction in rhapontigenin isomer 1 implies perturbed stilbenoid metabolism, potentially affecting inflammatory and immune response [58], while diminished 6-hydroxykynurenic acid and pyrrolidine in the EFA2 group highlight shifts in tryptophan and pyrrolizidine-alkaloid pathways that may modulate neurological signaling and feed-intake control [59].

Conversely, elevated cyanidin 3-*O*-glucoside-7-*O*-(6-*O*-(p-hydroxybenzoyl)-glucoside) and related anthocyanins denote enhanced flavonoid metabolism, potentially boosting antioxidant capacity and dampening inflammatory processes in both the rumen and systemic circulation [48]. Higher concentrations of prolyl-aspartate, histidine, and other amino-acid derivatives further suggest improved protein catabolism and nitrogen capture, favoring greater metabolizable-protein flow and milk-protein synthesis [40]. Histidine plays a vital role in various bodily functions, including serving as a precursor for histamine synthesis, metal chelation, pH buffering, and immune function [60]. Detection of specialized nitrogenous metabolites (e.g., 2-aminovalienone, 2-aminophenol, 5-aminopentanoic acid) further underscores a metabolically versatile microbial community capable of generating bioactive, antimicrobial compounds that reinforce an anti-methanogenic milieu. Finally, the rise in guaiacol and coniferyl-aldehyde isomer 1 indicates intensified lignin-derived phenolic turnover, which may alter plant-cell-wall interactions with fibrolytic microbes and thereby modulate fiber digestion [32]. The accompanying changes in phenolic derivatives such as 3,4-dihydroxyphenylvaleric acid isomer 1 and 5-hydroxyconiferyl alcohol signal broader polyphenol-metabolism adjustments that could influence rumen redox balance and microbial protein synthesis [9]. The comprehensive metabolite shifts observed with EFA2 align with previous reports on oregano and clove oils, which contain carvacrol, thymol, and eugenol—compounds known to modify rumen fermentation through selective antimicrobial activity against Gram-positive bacteria and methanogens [9,37].

The observed enrichment in pyrimidine and purine metabolism points to enhanced nucleic acid turnover and microbial proliferation, likely contributing to improved microbial protein synthesis and nitrogen capture [56]. The concurrent activation of biotin metabolism suggests improved cofactor availability for essential carboxylation reactions, which play a central role in fatty acid synthesis and energy metabolism. Furthermore, the upregulation of nitrogen, alanine, aspartate, and glutamate metabolism alongside biosynthetic pathways for arginine, phenylalanine, tyrosine, and tryptophan indicates a comprehensive enhancement of amino acid biosynthesis and turnover. Moreover, modulation of amino acid and nucleotide signaling pathways may influence microbial community structure and functional resilience, contributing to a rumen environment less conducive to methanogenesis and more efficient in nutrient extraction.

The EFA3 treatment, containing clove, anise, peppermint, and oregano with fumaric acid, produced distinctive metabolite alterations characterized by significant increases in pyrocatechol and taurine (2.91-fold), ethanolamine (2.73-fold), and creatine (2.22-fold). The marked elevation in pyrocatechol (a phenolic compound) suggests enhanced antimicrobial activity particularly targeting methanogens, as phenolic compounds can disrupt microbial cell membranes and inhibit specific enzymes involved in methanogenesis [9]. The substantial increase in taurine is particularly relevant to CH_4_ mitigation, as sulfur-containing amino acids have been shown to compete with methanogenesis for hydrogen utilization [41]. Notably, the elevated taurine concentration in the EFA3 treatment may also benefit host metabolism, given taurine’s roles in enhancing lipid metabolism and reducing inflammation [61].

Ethanolamine, a naturally occurring amino alcohol and component of phosphatidylethanolamine, plays a multifaceted role in ruminant physiology and microbial ecology. In the rumen, ethanolamine serves as a carbon and nitrogen source for certain bacteria (e.g., *Clostridium*, *Enterococcus*), which metabolize it via the *eut* operon into acetaldehyde and ammonia thus fueling microbial protein synthesis and VFA production [62,63]. Importantly, ethanolamine catabolism requires less hydrogen than traditional fermentative pathways, offering a competitive sink for H_2_ and potentially reducing enteric CH_4_ emissions [64]. Beyond its microbial role, ethanolamine is also a precursor to *N*-acylethanolamines (NAEs), which modulate immune function and gut integrity, contributing to animal health [65]. Industrially, ethanolamine’s CO_2_-binding properties suggest potential for integration into manure or biogas management strategies to further mitigate greenhouse gas emissions.

Additionally, the upregulation of creatine suggests potential improvements in energy metabolism efficiency, which could support more effective energy partitioning in the host. Ethanolamine upregulation may indicate phospholipid metabolism alterations that could affect microbial membrane integrity, particularly in methanogens which are sensitive to changes in membrane fluidity [66]. The increase in 3′,5′-cyclic GMP (1.86-fold) in EFA3, similar to that observed in EFA2, further supports altered microbial signaling pathways that may influence population dynamics.

Another notable finding from the PLS-DA scores is the distinctive metabolic signature of the EFA3 treatment, characterized by enhanced dipeptide formation and specialized metabolism of aromatic compounds. Lysyl-tyrosine emerged as a particularly significant biomarker, indicative of improved protein metabolism and amino acid utilization efficiency. Peptides and amino acids, whether directly supplied through the diet or released via proteolysis in the rumen, serve as key nutrients for microbial growth [67,68]. The increased concentration of lysyl-tyrosine suggests elevated proteolytic activity and more efficient peptide transport mechanisms within the rumen ecosystem. In ruminants, methionine and lysine are typically the first limiting amino acids for growth and production [69,70], making the detection of lysine-containing dipeptides such as lysyl-tyrosine particularly valuable for supporting optimal metabolic function. Furthermore, the presence of lysyl-tyrosine also reflects enhanced aromatic amino acid metabolism, as rumen microbes can synthesize tyrosine from phenylalanine [71], indicating that the EFA3 treatment may have promoted more efficient amino acid interconversion pathways.

The elevated concentrations of prolyl-aspartate and prolyl-valine further emphasize the enhanced dipeptide metabolism under EFA3 treatment, with these compounds serving as important indicators of active protein turnover and amino acid recycling within the rumen environment. Valine is oxidatively deaminated and decarboxylated by rumen microorganisms in vitro giving rise to isobutyric acid, and the formation of prolyl-valine suggests that the EFA3 treatment redirected valine metabolism toward peptide formation rather than complete deamination, potentially improving nitrogen retention efficiency [72]. The enrichment of proline-containing dipeptides such as prolyl-aspartate and prolyl-valine is particularly significant as proline catabolism via proline oxidase/proline dehydrogenase responds to nutrient and environmental stress by supplying energy, regulating redox balance, and facilitating osmoprotection [73]. These findings imply that EFA3 may bolster the rumen’s adaptive capacity to fluctuating nutritional or environmental conditions by supporting proline-mediated homeostatic mechanisms.

The reappearance of 3-sulfocatechol in the EFA3 metabolite profile, similar to the EFA1 treatment, indicates consistent enhancement of phenolic compound metabolism and sulfation pathways across different essential oil formulations [47]. This sulfated catechol derivative represents active phase II detoxification processes and suggests that the EFA3 treatment maintained the beneficial effects on antioxidant metabolism while potentially offering additional metabolic advantages through its unique dipeptide profile [26,48]. The presence of complex metabolites such as 4-(2-amino-3-hydroxyphenyl)-2,4-dioxobutanoic acid and the various hydroxy-dienedioic acid derivatives indicates sophisticated biochemical transformations involving aromatic amino acid catabolism and fatty acid metabolism, suggesting that the EFA3 treatment promoted metabolic flexibility and enhanced the rumen’s capacity to process diverse substrates [74].

The detection of asperxanthone, a fungal-derived anthraquinone compound, represents a unique aspect of the EFA3 metabolic profile and suggests the presence of specialized microbial populations capable of producing bioactive secondary metabolites with potential antimicrobial and antioxidant properties [75]. This compound’s presence indicates that the EFA3 treatment may have selectively promoted certain fungal or actinobacterial populations within the rumen microbiome, contributing to enhanced metabolic diversity and potentially supporting the anti-methanogenic effects through the production of specialized bioactive compounds. The overall metabolite signature of EFA3 treatment demonstrates a sophisticated balance between enhanced protein metabolism, improved nitrogen utilization, maintained antioxidant capacity, and the production of unique bioactive compounds, suggesting that this formulation offers a comprehensive approach to optimizing rumen function while supporting CH_4_ mitigation strategies.

The enrichment of KEGG pathways including lipoic acid, porphyrin, glyoxylate and dicarboxylate, nitrogen, glycine-serine-threonine, taurine-hypotaurine metabolism, and primary bile acid biosynthesis in response to EFA3 supplementation suggests a broad enhancement of metabolic adaptability and nutrient utilization in the rumen. Lipoic acid, a key mitochondrial cofactor, is vital for oxidative decarboxylation reactions, supporting energy metabolism under stress or nutrient-limited conditions [76]. Enrichment in porphyrin metabolism indicates upregulated heme biosynthesis, essential for microbial electron transport and oxygen-sensitive enzyme systems, which are particularly relevant in anaerobic rumen fermentation. Glyoxylate and dicarboxylate pathway activation suggests improved gluconeogenic potential from non-carbohydrate substrates, complementing ethanolamine and proline metabolism that also fuel microbial energy and nitrogen assimilation [62]. Enhanced nitrogen metabolism further aligns with observed increases in dipeptides such as glycyl-prolyl-hydroxyproline, supporting microbial protein synthesis and efficient nitrogen capture. Additionally, the upregulation of taurine, hypotaurine, and bile acid biosynthesis pathways may indicate modulation of microbial-host interactions, potentially improving gut integrity and anti-inflammatory responses [65]. These pathway enrichments reflect EFA3’s capacity to promote a metabolically flexible and environmentally efficient rumen microbiome.

Several metabolites showed consistent directional changes across all three EFA treatments, suggesting common mechanistic pathways despite the different essential oil compositions. The universal downregulation of phenylpropanolamine across all treatments (0.37-fold, 0.47-fold, and 0.39-fold in EFA1, EFA2, and EFA3, respectively) indicates a fundamental shift in catecholamine metabolism. The significant downregulation of *N*,*N*-didesmethyltramadol (0.27-fold) and phenylpropanolamine (0.39-fold) across all three EFA treatments suggests consistent effects on alkaloid metabolism regardless of essential oil blend composition. This consistency suggests that the combination of essential oils with fumaric acid may target specific pathways regardless of the exact essential oil composition. The consistent downregulation of 8-aminooctanoic acid across treatments may indicate altered fatty acid metabolism with potential implications for milk fat synthesis. Previous studies have demonstrated that essential oils can modulate biohydrogenation pathways, potentially affecting milk fatty acid profiles [77].

The fumaric acid component likely functioned as an alternative hydrogen sink, competitively reducing hydrogen availability for methanogenesis through conversion to succinate and ultimately propionate [78]. This process would theoretically capture reducing equivalents that would otherwise be utilized for CH_4_ formation, thereby reducing methanogenesis while enhancing energy retention as propionate [79]. The combined effect between essential oils and fumaric acid may explain the substantial metabolite alterations observed. Essential oil components likely disrupt methanogen membrane integrity and inhibited specific enzymes involved in methanogenesis [37], while fumaric acid simultaneously provided an alternative metabolic hydrogen sink [17].

The observed metabolite alterations suggest broader implications for rumen function and host metabolism. The upregulation of phenolic compounds (isoferulic acid, guaiacol, pyrocatechol) across treatments indicates enhanced antioxidant capacity within the rumen environment, which may mitigate oxidative stress and improve epithelial health [80]. These metabolite alterations suggest that EFA combinations not only target methanogenesis but may also improve overall fermentation efficiency and nutrient utilization.

## 5. Conclusions

The mixture of unique essential oil blends and fumaric acid revealed distinct metabolite profiles and key metabolic pathways linked to energy and nutrient utilization, methanogenesis and overall rumen function. EFA1 treatment led to the enrichment of tyrosine, purine, pyrimidine, lipoic acid, porphyrin, and phosphate metabolism pathways. EFA2 supplementation enriched key metabolic pathways, including pyrimidine, purine, biotin, and nitrogen metabolism, as well as the metabolism of alanine, aspartate, and glutamate, and the biosynthesis of arginine, phenylalanine, tyrosine, and tryptophan. EFA3 supplementation resulted in the enrichment of multiple metabolic pathways, including those related to lipoic acid, porphyrin, glyoxylate and dicarboxylate metabolism, nitrogen cycling, glycine, serine, and threonine metabolism, taurine and hypotaurine metabolism, and primary bile acid biosynthesis. These additives have the potential to optimize nutrient utilization and overall animal health. Therefore, an in vivo study should be conducted to validate their efficacy.

## Figures and Tables

**Table 1:** Fold change in metabolites between EFA1 and CON group.

Metabolites	Fold Change	Log2(FC)	FDR
Taurine	2.4752	1.3075	0.0110
Isomer 2 of 2,7-Dihydroxycadalene	2.2466	1.1678	0.0110
Isoferulic acid	1.7146	0.77787	0.0134
Pyrocatechol	1.6607	0.73178	0.0151
Guaiacol	1.6553	0.72708	0.0151
Isomer 1 of 2,4-Diamino-6-hydroxypyrimidine	1.6304	0.70524	0.0151
1*H*-Indole-3-methanamine	1.5707	0.65137	0.0151
Seryl-Tyrosine	1.5119	0.59634	0.0163
3′,5′-Cyclic GMP	1.5049	0.58971	0.0163
2,8-Dihydroxyadenine	0.65687	−0.60632	0.0151
Piceatannol	0.65434	−0.61189	0.0151
2-hydroxymexiletine	0.65078	−0.61976	0.0163
Isomer 2 of Homoeriodictyol chalcone	0.64859	−0.62462	0.0163
4-Aminobutanoic acid	0.63386	−0.65777	0.0165
Litcubinine	0.62962	−0.66744	0.0168
Citrulline	0.61313	−0.70573	0.0172
Desoxyhemigossypol-6-Methyl Ether	0.60158	−0.73317	0.0183
Isomer 1 of Homoeriodictyol chalcone	0.56125	−0.83328	0.0185
Calystegin A3	0.53417	−0.90462	0.0188
*N*,*N*-Didesmethyltramadol	0.51099	−0.96864	0.0191
8-Aminooctanoic acid	0.50719	−0.9794	0.0193
5-Aminolevulinic acid	0.49522	−1.0139	0.0213
Dopamine 4-*O*-glucuronide	0.44884	−1.1557	0.0213
Phenylpropanolamine	0.36712	−1.4457	0.0240
Isomer 1 of 2,6-Dimethyl-1,4-benzenediol	0.36263	−1.4634	0.0498

EFA1, received essential oil blend (Garlic, Lemongrass, Cumin, Lavender, and Nutmeg at 4:2:2:1:1, respectively) and fumaric acid; CON, control group.

**Table 2:** Fold change in metabolites between EFA2 and CON group.

Metabolites	Fold Change	Log2(FC)	FDR
4-Hydroxy-5-(dihydroxyphenyl)-valeric acid-*O*-sulphate	2.5864	1.371	0.0003
3′,5′-Cyclic GMP	2.3826	1.2525	0.0005
Guaiacol	2.1393	1.0972	0.0015
5-Methoxytryptophan	1.9607	0.97139	0.0066
Isomer 1 of 2-Hydroxyhepta-2,4-dienedioic acid	1.9465	0.96086	0.0079
Creatine	1.9034	0.92855	0.0096
2,3-Diaminoproprionic acid	1.8573	0.89323	0.0020
Isomer 1 of 2-Aminovalienone	1.85	0.88752	0.0023
Aspartic acid	1.8313	0.87285	0.0027
Proline	1.8013	0.84902	0.0035
Isomer 1 of Morpholine	1.7777	0.82999	0.0042
Norfuraneol	1.7633	0.81827	0.0046
2-Aminoethylphosphonate	1.7554	0.81178	0.0047
Chlorohydroquinone	1.7399	0.79898	0.0049
Rubroskyrin	1.7387	0.79799	0.0049
2,3-Diaminosalicylic acid	1.7156	0.77871	0.0053
4-Guanidinobutanal	1.7100	0.77401	0.0055
Isomer 1 of 3-Cyano-l-alanine	1.6767	0.74563	0.0056
(+/−)-*N*-Methylsalsolinol	1.6728	0.74225	0.0056
*N*2-Acetyl-5-Phosphooxy-l-lysine	1.6661	0.73647	0.0057
4-Fluorocatechol	1.6518	0.72408	0.0057
2-Hydroxy-6-oxonona-2,4-diene-1,9-dioic acid	1.6486	0.72127	0.0061
1*H*-Indole-3-methanamine	1.6378	0.71177	0.0064
Isomer 2 of Seryl-Valine	1.6353	0.70955	0.0067
2-Methylbutylamine	1.6281	0.70317	0.0068
1-Methylguanosine	1.6266	0.7019	0.0078
Isomer 1 of Hulupinic acid	1.6238	0.69941	0.0101
Isomer 1 of 3-Hydroxyaminophenol	1.6237	0.69928	0.0127
*N*2-Acetyl-*N*6-(l-1,3-Dicarboxypropyl)-l-lysine	1.6086	0.68583	0.0139
Glycine	1.5659	0.64698	0.0139
l-*trans*-4-Methyl-2-pyrrolidinecarboxylic acid	1.5658	0.64693	0.0161
*N*6-beta-Aspartyl-Lysine	1.5638	0.64509	0.0165
4-Formylsalicylic acid	1.5592	0.64081	0.0188
(3*S*,5*S*)-3,5-Diaminohexanoic acid	1.5559	0.63774	0.0200
Taurine	1.5505	0.63276	0.0202
Ethanolamine	1.5503	0.6325	0.0213
Lysyl-Tyrosine	1.5474	0.62989	0.0215
Isomer 1 of 5-Aminopentanoic acid	1.5316	0.61501	0.0218
4-Aminobutyraldehyde	1.5307	0.6142	0.0221
4-Hydroxypiperidine	1.5275	0.6112	0.0243
Isomer 2 of 2,7-Dihydroxycadalene	1.5207	0.60475	0.0334
Sarcosine	1.5200	0.60405	0.0334
Seryl-Tyrosine	1.5196	0.60373	0.0351
Ascorbic acid	1.5195	0.60358	0.0368
*N*(6)-Methyllysine	1.5125	0.59693	0.0373
Aspartyl-Proline	1.5101	0.59462	0.0401
Isomer 1 of Lysyl-Glutamate	1.5082	0.59281	0.0432
Lysyl-Valine	0.66326	−0.59235	0.0002
dl-2-Aminooctanoic acid	0.65867	−0.60238	0.0003
2,4-Diamino-6-hydroxypyrimidine	0.65579	−0.6087	0.0004
2-Aminomuconic acid	0.64948	−0.62265	0.0004
1,4-diaminobutane	0.64692	−0.62833	0.0005
Alanyl-Proline	0.63768	−0.6491	0.0006
Kuwanol D	0.63195	−0.66212	0.0006
Alanyl-Lysine	0.62479	−0.67856	0.0007
Isomer 1 of Homoeriodictyol chalcone	0.62326	−0.6821	0.0007
3-Hydroxyaminophenol	0.62162	−0.6859	0.0008
6-Hydroxykynurenic acid	0.62068	−0.68809	0.0010
Valyl-Valine	0.61277	−0.70659	0.0013
Citrulline	0.61247	−0.7073	0.0015
Isomer 1 of Monodehydroascorbic acid	0.61032	−0.71236	0.0015
Glutamyl-Leucine	0.61024	−0.71256	0.0027
3-Formyl-6-hydroxyindole	0.60358	−0.72837	0.0057
Leucyl-Valine	0.59776	−0.74237	0.0057
Litcubinine	0.59736	−0.74333	0.0078
Isoleucyl-Lysine	0.59598	−0.74667	0.0101
Threoninyl-Valine	0.59453	−0.75019	0.0127
8-Aminooctanoic acid	0.56984	−0.81136	0.0139
Calystegin A3	0.56768	−0.81685	0.0139
Seryl-Valine	0.56261	−0.8298	0.0161
Adenine	0.56078	−0.83449	0.0165
2,6-Dimethoxy-4-methylphenol	0.55841	−0.84062	0.0188
Isomer 1 of Rhapontigenin	0.53274	−0.90849	0.0200
Leucyl-Leucine	0.48451	−1.0454	0.0221
Phenylpropanolamine	0.47098	−1.0863	0.0243
4-Aminobutanoic acid	0.45787	−1.127	0.0334
5-Aminolevulinic acid	0.45439	−1.138	0.0334
Dopamine 4-*O*-glucuronide	0.41192	−1.2796	0.0351
Isomer 1 of 2,6-Dimethyl-1,4-benzenediol	0.39663	−1.3341	0.0373
*N*,*N*-Didesmethyltramadol	0.21681	−2.2055	0.0401

EFA2, received essential oil blend (Anise, Clove, Oregano, Cedarwood, and Ginger at 4:2:2:1:1, respectively) and fumaric acid; CON, control group.

**Table 3:** Fold change in metabolites between EFA3 and CON group.

Metabolites	Fold Change	Log2(FC)	FDR
Pyrocatechol	2.9137	1.5428	0.0020
Taurine	2.9068	1.5394	0.0023
Ethanolamine	2.7283	1.448	0.0023
Creatine	2.2189	1.1498	0.0027
*N*,*N*,*O*-Tridesmethyltramadol	2.1277	1.0893	0.0035
1-Methylguanosine	1.997	0.99785	0.0056
(+/−)-*N*-Methylsalsolinol	1.9487	0.96248	0.0056
Indoline-2-carboxylic acid	1.9400	0.95608	0.0083
Isomer 1 of Morpholine	1.9090	0.93285	0.0096
3′,5′-Cyclic GMP	1.8645	0.89878	0.0101
Isomer 1 of (2*R*,3*R*,4*R*)-2-Amino-4-hydroxy-3-methylpentanoic acid	1.6542	0.72614	0.0127
Isomer 2 of 2,7-Dihydroxycadalene	1.6274	0.70261	0.0150
Tyramine	1.5818	0.66161	0.0178
1*H*-Indole-3-methanamine	1.5545	0.63645	0.0191
2-Isopropyl-1,4-benzenediol	1.5507	0.63296	0.0223
2-Phenylethylamine	1.5339	0.6172	0.0223
Threoninyl-Threonine	1.5278	0.61145	0.0256
Methylguanidine	1.5261	0.60987	0.0278
2,6-Dimethyl-1,4-benzenediol	1.5086	0.59324	0.0284
Isomer 2 of Homoeriodictyol chalcone	0.6653	−0.58792	0.0064
Eugenol	0.6641	−0.59053	0.0083
Litcubinine	0.66344	−0.59196	0.0096
Isomer 1 of Pyridoxamine	0.65486	−0.61074	0.0101
Sinapyl alcohol	0.65398	−0.61267	0.0127
2-Aminomuconic acid	0.64724	−0.62762	0.0150
2-hydroxymexiletine	0.64141	−0.64068	0.0178
Dulciol E	0.6358	−0.65335	0.0191
5-Hydroxyxanthotoxin	0.62955	−0.6676	0.0223
2,3,6-Trihydroxypyridine	0.62115	−0.68699	0.0223
Isomer 1 of Dihydroconiferyl Alcohol	0.61012	−0.71283	0.0256
Desoxyhemigossypol-6-Methyl Ether	0.60952	−0.71426	0.0278
Piceatannol	0.60926	−0.71488	0.0284
Calystegin A3	0.60395	−0.72749	0.0313
5-Aminolevulinic acid	0.59936	−0.73849	0.0320
Isomer 4 of (−)-Vestitol	0.53257	−0.90896	0.0337
Isomer 1 of 2,6-Dimethyl-1,4-benzenediol	0.44556	−1.1663	0.0361
Phenylpropanolamine	0.38726	−1.3686	0.0365
*N*,*N*-Didesmethyltramadol	0.2716	−1.8805	0.0430

EFA3, received essential oil blend (Clove, Anise, Peppermint, and Oregano at 4:3:2:1, respectively) and fumaric acid; CON, control group.

**Table 4:** PLS-DA cross-validation (5-fold CV) of latent components.

Treatments *	Measure	1 Comp	2 Comps	3 Comps	4 Comps
EFA1	Accuracy	1.0000	1.0000	1.0000	1.0000
	R^2^	0.99878	0.99882	0.99886	0.99981
	Q^2^	0.56579	0.8953	0.99254	0.9638
EFA2	Accuracy	1.0000	1.0000	1.0000	1.0000
	R^2^	0.96769	0.99887	0.9989	0.9998
	Q^2^	0.71056	0.91829	0.99026	0.9720
EFA3	Accuracy	1.0000	1.0000	1.0000	1.0000
	R^2^	0.99447	0.99798	0.99815	0.99898
	Q^2^	0.59845	0.89773	0.98844	0.9387

* Footnotes on EFA1, EFA2 and EFA3 are shown in Table 1, Table 2 and Table 3 above.

**Table 5:** KEGG-based pathway enrichment analysis of metabolites altered by EFA1 treatment.

Pathway Name	Total Metabolites	Hits	Impact Value	*p*-Value	FDR
Tyrosine metabolism	42	13	0.310	0.0001	5.74 × 10^−6^
Purine metabolism	70	11	0.157	0.0013	1.06 × 10^−4^
Pyrimidine metabolism	39	6	0.154	0.0048	5.79 × 10^−4^
Phosphonate and phosphinate metabolism	6	1	0.167	0.0087	0.0014
Histidine metabolism	16	6	0.375	0.0110	0.0022
Lipoic acid metabolism	28	1	0.036	0.0117	0.0028
Porphyrin metabolism	31	3	0.097	0.0134	0.0037
Cysteine and methionine metabolism	33	5	0.152	0.0192	0.0061
Arginine biosynthesis	14	6	0.429	0.0221	0.0080
Nitrogen metabolism	6	2	0.333	0.0234	0.0094
Glyoxylate and dicarboxylate metabolism	31	4	0.129	0.0250	0.0110
Glycine, serine and threonine metabolism	33	9	0.273	0.0267	0.0128
Alanine, aspartate and glutamate metabolism	28	6	0.214	0.0313	0.0163
Tryptophan metabolism	41	6	0.146	0.0414	0.0232
Glutathione metabolism	28	6	0.214	0.0415	0.0249
Primary bile acid biosynthesis	46	2	0.043	0.0417	0.0267
Butanoate metabolism	15	2	0.133	0.0418	0.0284
Pantothenate and CoA biosynthesis	20	6	0.300	0.0419	0.0302
Nicotinate and nicotinamide metabolism	15	1	0.067	0.0421	0.0320
Selenocompound metabolism	20	1	0.050	0.0422	0.0338
Arginine and proline metabolism	36	15	0.417	0.0423	0.0356
beta-Alanine metabolism	21	7	0.333	0.0532	0.0468
Propanoate metabolism	21	1	0.048	0.0792	0.0697
Taurine and hypotaurine metabolism	8	1	0.125	0.0800	0.0736
Ubiquinone and other terpenoid-quinone biosynthesis	18	3	0.167	0.0817	0.0785

**Table 6:** KEGG-based pathway enrichment analysis of metabolites altered by EFA2 treatment.

Pathway Name	Total Metabolites	Hits	Impact Value	*p*-Value	FDR
Pyrimidine metabolism	39	6	0.154	2.90 × 10^−5^	1.94 × 10^−7^
Purine metabolism	70	11	0.157	6.59 × 10^−4^	8.79 × 10^−5^
Biotin metabolism	10	1	0.100	0.00708	0.00142
Nitrogen metabolism	6	2	0.333	0.00993	0.00265
Arginine biosynthesis	14	6	0.429	0.01091	0.00364
Alanine, aspartate and glutamate metabolism	28	6	0.214	0.01291	0.00516
Phenylalanine, tyrosine and tryptophan biosynthesis	4	2	0.500	0.01766	0.00824
Porphyrin metabolism	31	3	0.097	0.02781	0.01483
Valine, leucine and isoleucine degradation	39	2	0.051	0.03321	0.01993
Histidine metabolism	16	6	0.375	0.03701	0.02467
Phosphonate and phosphinate metabolism	6	1	0.167	0.04099	0.03006
Glyoxylate and dicarboxylate metabolism	31	4	0.129	0.04695	0.03756
β-Alanine metabolism	21	7	0.333	0.04912	0.04257
Nicotinate and nicotinamide metabolism	15	1	0.067	0.05245	0.04895
Phenylalanine metabolism	8	3	0.375	0.05563	0.05563
Arginine and proline metabolism	36	15	0.417	0.05585	0.05957
Valine, leucine and isoleucine biosynthesis	8	3	0.375	0.06948	0.07874
Glutathione metabolism	28	6	0.214	0.07261	0.08713
Lipoic acid metabolism	28	1	0.036	0.07442	0.09427
Butanoate metabolism	15	2	0.133	0.07483	0.09977
Selenocompound metabolism	20	1	0.050	0.08941	0.12517
Cysteine and methionine metabolism	33	5	0.152	0.09789	0.14357
Glycine, serine and threonine metabolism	33	9	0.273	0.10412	0.15965
Pantothenate and CoA biosynthesis	20	6	0.300	0.10601	0.16962
Ubiquinone and other terpenoid-quinone biosynthesis	18	3	0.167	0.11376	0.18960

**Table 7:** KEGG-based pathway enrichment analysis of metabolites altered by EFA3 treatment.

Pathway Name	Total Metabolites	Hits	Impact Value	*p*-Value	FDR
Lipoic acid metabolism	28	1	0.036	6.11 × 10^−4^	2.54 × 10^−5^
Primary bile acid biosynthesis	46	2	0.043	6.94 × 10^−4^	5.78 × 10^−5^
Porphyrin metabolism	31	3	0.097	3.76 × 10^−4^	4.70 × 10^−5^
Glyoxylate and dicarboxylate metabolism	31	4	0.129	7.28 × 10^−4^	1.21 × 10^−4^
Nitrogen metabolism	6	2	0.333	9.44 × 10^−4^	1.97 × 10^−4^
Glycine, serine and threonine metabolism	33	9	0.273	0.0014	3.48 × 10^−4^
Pyrimidine metabolism	39	6	0.154	0.0020	5.94 × 10^−4^
Purine metabolism	70	11	0.157	0.0035	0.0012
Taurine and hypotaurine metabolism	8	1	0.125	0.0078	0.0029
Pantothenate and CoA biosynthesis	20	6	0.300	0.0233	0.0097
beta-Alanine metabolism	21	7	0.333	0.0288	0.0132
Phenylalanine, tyrosine and tryptophan biosynthesis	4	2	0.500	0.0304	0.0152
Lysine degradation	30	4	0.133	0.0430	0.0233
Cysteine and methionine metabolism	33	5	0.152	0.0482	0.0281
Glycerophospholipid metabolism	36	2	0.056	0.0484	0.0302
Ubiquinone and other terpenoid-quinone biosynthesis	18	3	0.167	0.0547	0.0365
Tryptophan metabolism	41	6	0.146	0.0563	0.0399
Phenylalanine metabolism	8	3	0.375	0.0597	0.0448
Tyrosine metabolism	42	13	0.310	0.0662	0.0524
Valine, leucine and isoleucine degradation	39	2	0.051	0.0702	0.0585
Histidine metabolism	16	6	0.375	0.0862	0.0755
Alanine, aspartate and glutamate metabolism	28	6	0.214	0.0897	0.0822
Arginine biosynthesis	14	6	0.429	0.0900	0.0863
Biotin metabolism	10	1	0.100	0.0981	0.0981
Valine, leucine and isoleucine biosynthesis	8	3	0.375	0.2143	0.2232
Selenocompound metabolism	20	1	0.050	0.2747	0.2976
Butanoate metabolism	15	2	0.133	0.2784	0.3131
Arginine and proline metabolism	36	15	0.417	0.2800	0.3266
Nicotinate and nicotinamide metabolism	15	1	0.067	0.2836	0.3426
Glutathione metabolism	28	6	0.214	0.3063	0.3829
Propanoate metabolism	21	1	0.048	0.4600	0.5942
Vitamin B6 metabolism	9	3	0.333	0.4922	0.6563
Phosphonate and phosphinate metabolism	6	1	0.167	0.8493	1.1678
D-Amino acid metabolism	15	1	0.067	0.8959	1.2692
Sphingolipid metabolism	32	1	0.031	0.8959	1.3066

## Data Availability

Data are contained within the article. Supplementary Data are also available in the repository.

## Supplementary Materials

The following supporting information can be downloaded at https://www.mdpi.com/article/10.3390/metabo15100681/s1: Figure S1: Box plot of samples of the metabolome between (A) EFA1 and CON group; (B) EFA2 and CON group; and (C) EFA3 and CON group before- and after- normalization; Figure S2: Permutation test showing the empirical values for both Q^2^ (*p* ≤ 0.01) and R^2^Y (*p* ≤ 0.01); Excel file: EFA Metabolome supplementary showing results of fold change, volcano plot and PLS-DA VIP Scores.
