# Supplementary material for: Untargeted Metabolomics of Dairy Cows as Influenced by the Combinations of Essential Oil Blends and Fumaric Acid as Natural Feed Additives Using RUSITEC
Source: Metabolites. 2025 Oct 21;15(10):681. doi: 10.3390/metabo15100681 (PMC12566130; doi:10.3390/metabo15100681)
Supplement: Supplementary file 1 [file metabolites-15-00681-s001.zip › EFA_Supplementary Figures.pdf]

Supplementary Figures/Materials

A

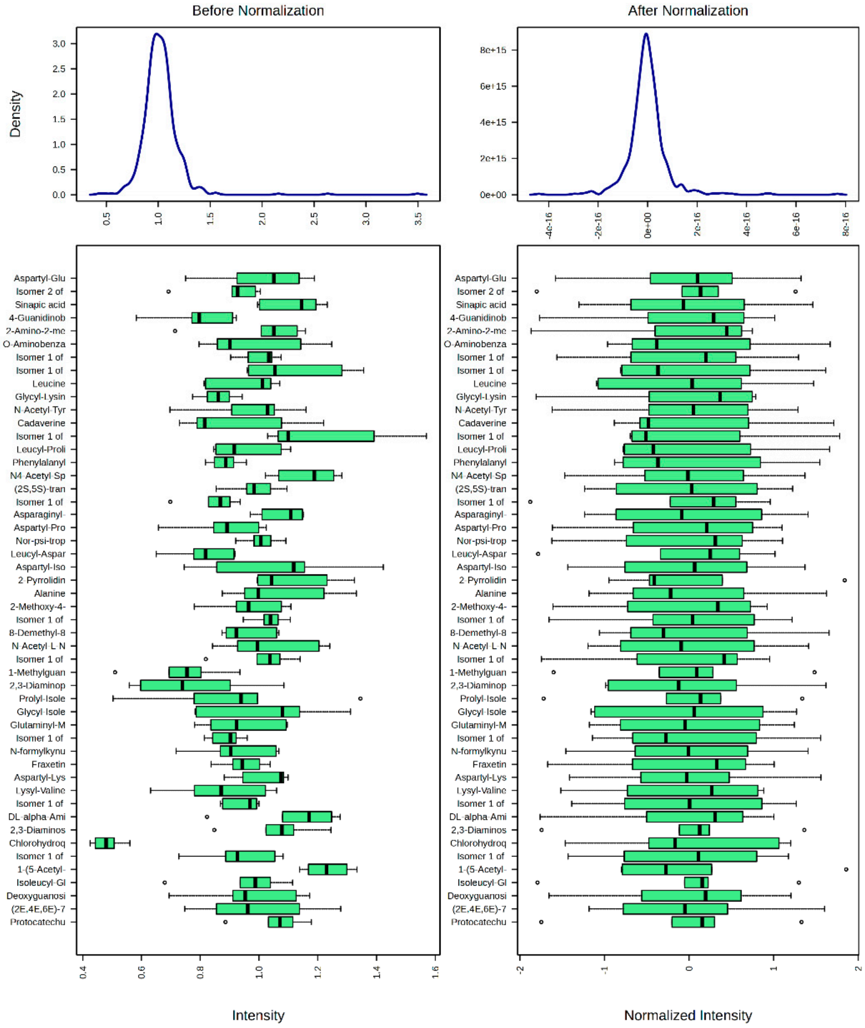

B

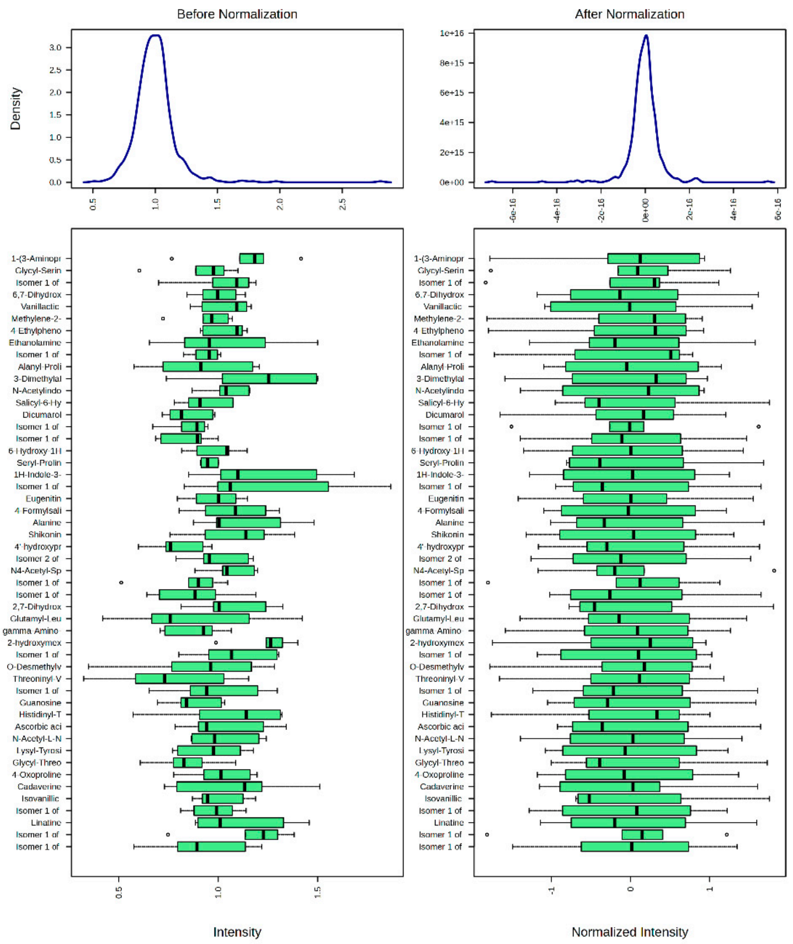
Before Normalization
After Normalization
Density
0.0
0.5
1.0
1.5
2.0
2.5
3.0
0e+00
2e+15
4e+15
6e+15
8e+15
1e+16
-6e-16
-4e-16
-2e-16
0e+00
2e-16
4e-16
6e-16
1-(3-Aminopr
Glycyl-Serin
Isomer 1 of
6,7-Dihydrox
Vanillactic
Methylene-2-
4-Ethylpheno
Ethanolamine
Isomer 1 of
Alanyl-Proli
3-Dimethylal
N-Acetylindo
Salicyl-6-Hy
Dicumarol
Isomer 1 of
Isomer 1 of
6-Hydroxy-1H
Seryl-Prolin
1H-Indole-3-
Isomer 1 of
Eugenitin
4-Formylsali
Alanine
Shikonin
4'-hydroxypr
Isomer 2 of
N4-Acetyl-Sp
Isomer 1 of
Isomer 1 of
2,7-Dihydrox
Glutamyl-Leu
gamma-Amino
2-hydroxymox
Isomer 1 of
O-Desmethylv
Threoninyl-V
Isomer 1 of
Guanosine
Histidinyl-T
Ascorbic aci
N-Acetyl-L-N
Lysyl-Tyrosi
Glycyl-Threo
4-Oxoproline
Cadaverine
Isovanillic
Isomer 1 of
Linatine
Isomer 1 of
Isomer 1 of
-1
0
1
Intensity
Normalized Intensity

C

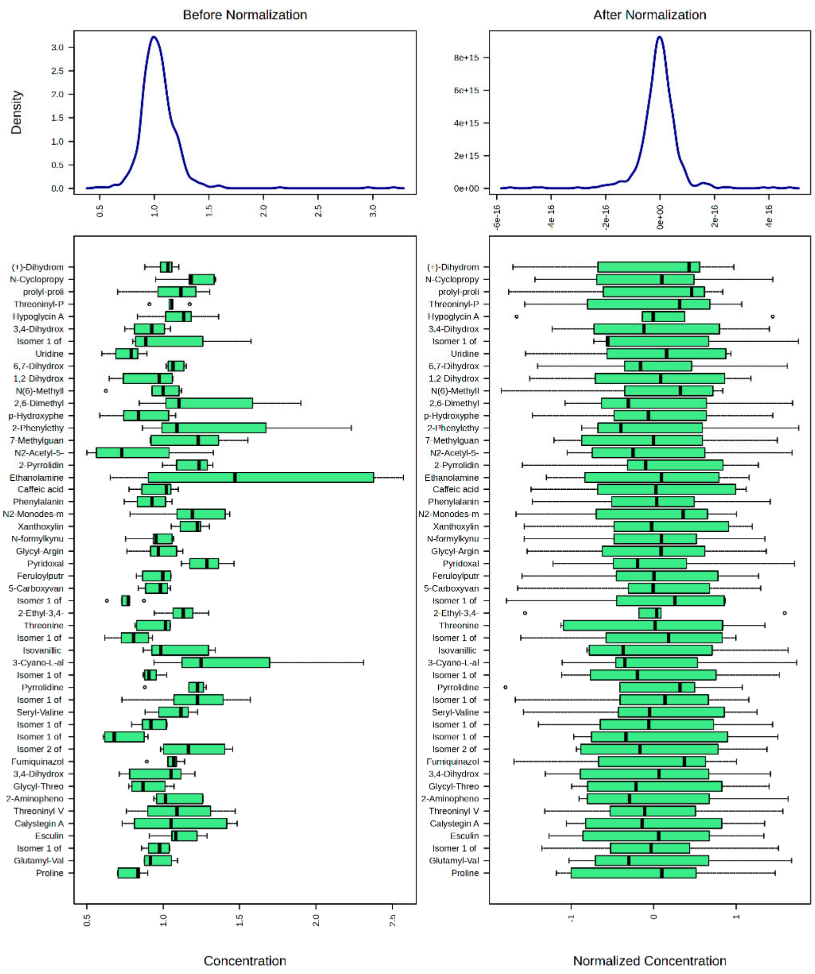

Figure S1. Box plot of samples of the metabolome between (A) EFA1 and CON group; (B) EFA2 and CON group; and (C) EFA3 and CON group before- and after- normalization.

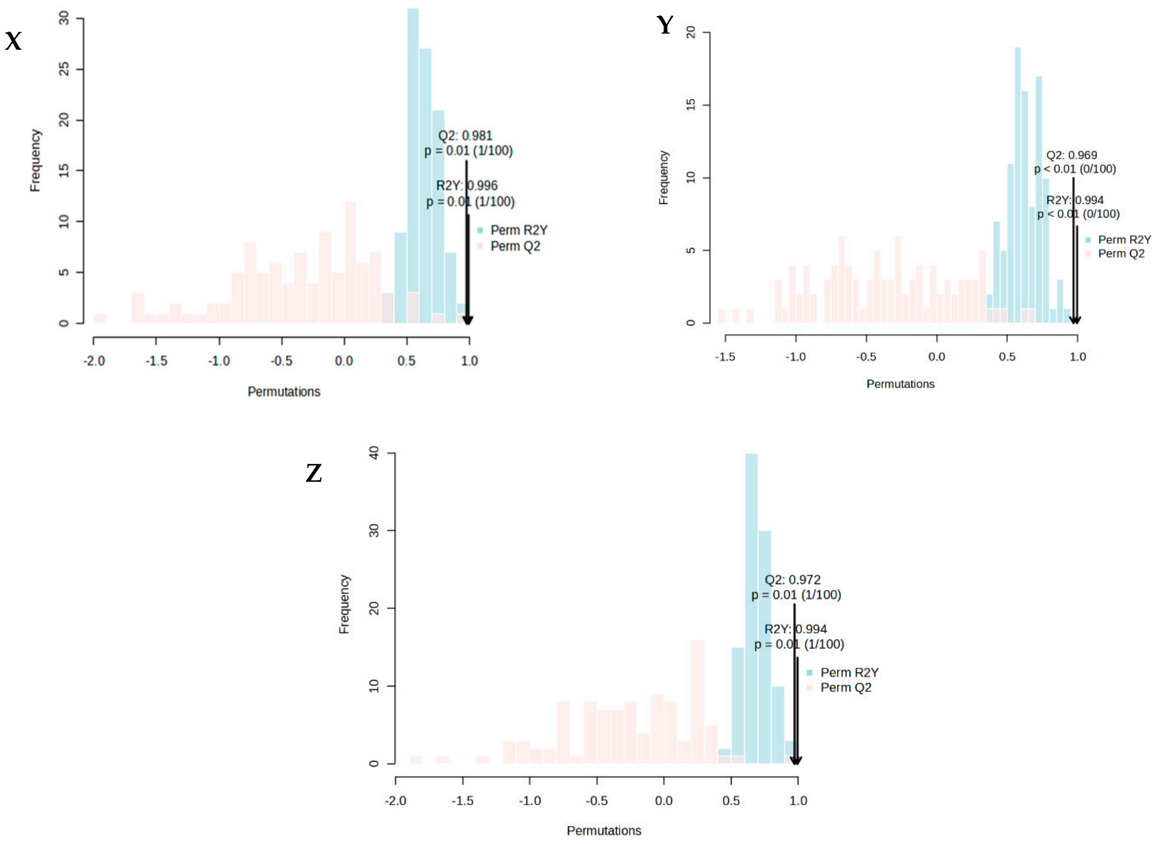

Figure S2. Permutation test showing the empirical values for (X) EFA1 group (Q^2: $p = 0.01$; R^2Y: $p = 0.01$); (Y) EFA2 group (Q^2: $p < 0.01$; R^2Y: $p < 0.01$); and (Z) EFA3 group (Q^2: $p = 0.01$; R^2Y: $p = 0.01$).
